# A fermented milk drink containing *Lactobacillus casei* strain Shirota modulates the esophageal microbiome composition in Barrett’s esophagus

**DOI:** 10.1016/j.isci.2026.116910

**Published:** 2026-07-22

**Authors:** Yonne Peters, Maria Laura Ferrando, Chengliang Zhou, Rene te Morsche, Britt van der Leeden, Renske Cremers, Phuc Dat Le, Leander van Dijk, Ruud W.M. Schrauwen, Adriaan C. Tan, Rachel S. van der Post, Peter van Baarlen, Peter D. Siersema, Annemarie Boleij

**Affiliations:** 1 Department of Gastroenterology and Hepatology, Radboudumc, Nijmegen, the Netherlands; 2 Host-Microbe Interactomics, Wageningen University and Research, Wageningen, the Netherlands; 3 Istituto di Ricerca Genetica e Biomedica (IRGB), Cagliari, Italy; 4 Department of Pathology, Radboudumc, Nijmegen, the Netherlands; 5 Department of Gastroenterology and Hepatology, Bernhoven Hospital, Uden, the Netherlands; 6 Department of Gastroenterology and Hepatology, Canisius Wilhelmina Hospital, Nijmegen, the Netherlands; 7 Department of Gastroenterology and Hepatology, Erasmus MC University Medical Center, Rotterdam, the Netherlands

**Keywords:** esophageal cancer, Barrett’s esophagus, microbiome, *Lactobacillus casei* Shirota, Yakult

## Abstract

Esophageal adenocarcinoma and its precursor, Barrett’s esophagus (BE), are associated with a pro-inflammatory, Gram-negative-dominated esophageal microbiome. In a single-arm pilot study, 23 patients with BE consumed a fermented milk drink containing *Lactobacillus casei* strain Shirota (LcS) twice daily for 4 weeks, based on the hypothesis that this intervention could shift the microbiome toward a more beneficial Gram-positive profile. Post-intervention, metaplastic columnar epithelium showed a significant increase in Gram-positive Firmicutes (*p* < 0.01) without changes in overall Eubacterial abundance. DNA-based analyses demonstrated a higher Gram-positive to Gram-negative ratio, with Proteobacteria decreasing from 74% to 52% and Firmicutes increasing from 20% to 31%, including enrichment of *Lactobacillus*. Microbial diversity increased markedly (*p* = 9.98 × 10^−7^) in squamous and metaplastic epithelium. Notably, BE-associated taxa *Prevotella* and *Haemophilus* also increased in both tissue types. Overall, the intervention shifted the esophageal microbiome toward a more Gram-positive and diverse composition, while highlighting complex ecological effects warranting further investigation.

## Highlights

• Barrett’s esophagus is associated with a Gram-negative, pro-inflammatory microbiome
• The fermented milk drink is able to modulate the esophageal microbiome
• Short-term intake increases Gram-positive taxa and microbial diversity
• These findings support microbiome-targeted clinical trials in Barrett’s esophagus

## Graphical abstract

**Figure:**
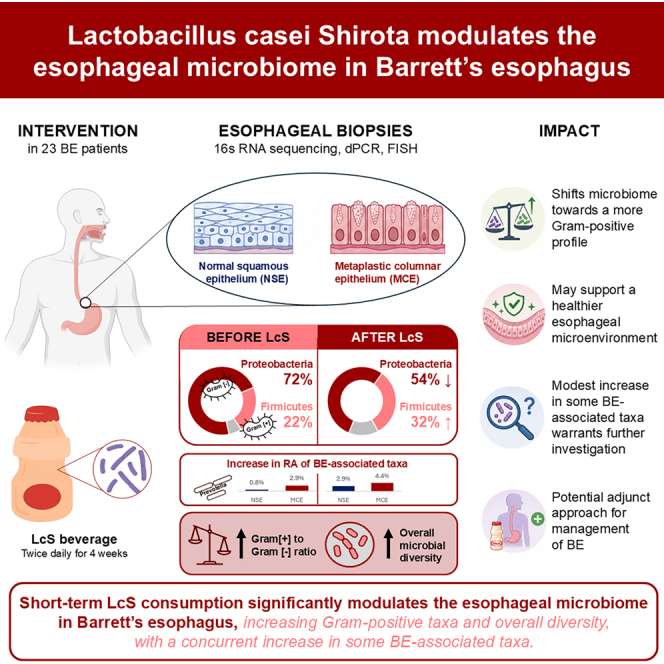

## Teaser

Microbiome; Cancer

## Introduction

The incidence of esophageal adenocarcinoma (EAC) has been rising rapidly in Western countries over the past few decades.^1^ Because EAC is frequently detected at an advanced stage, patients with EAC have a dismal prognosis with a five-year survival rate of less than 20%.^2^ The only known precursor of EAC is Barrett’s esophagus (BE), with patients with BE having a 30- to 125-fold increased risk of developing EAC.^3^ BE is characterized by the replacement of normal squamous epithelium (NSE) in the distal esophagus with metaplastic columnar epithelium (MCE). This metaplastic change is largely attributed to gastroesophageal reflux disease (GERD), affecting globally about 15%–20% of the general population,^4^ resulting in injury of the squamous esophageal mucosa. However, not all patients with GERD develop BE.^5^

While GERD is the main risk factor for BE, BE pathogenesis is complex and multifactorial. The microenvironment of BE is shaped by the inflammatory response due to chronic reflux resulting in the production of pro-inflammatory cytokines. At the same time, there is increasing evidence that the esophageal microbiome differs in patients with and without BE.^6^ Specific alterations in the abundances of specific taxa of the esophageal microbiome may contribute to the pathogenesis of BE and its progression to EAC through mechanisms including chronic inflammation and barrier dysfunction influencing the local microenvironment.^7^ Once thought to be minimal, the esophageal microbiome is now recognized as having a diverse and stable bacterial population.^8^ In healthy individuals, the esophagus is predominantly colonized by carbohydrate-degrading, short-chain fatty-acid (SCFA)-producing Gram-positive bacteria, especially taxa of the genus *Streptococcus*.^9^^,^^10^^,^^11^ In contrast, BE is characterized by an increase in the relative abundance (RA) of disease-associated Gram-negative bacteria^3^^,^^4^ (taxa from the genera *Fusobacterium*, *Veillonella*, *Prevotella*, *Haemophilus*, *Neisseria*, and *Campylobacter*^12^) and a decrease in RA of Gram-positive bacteria, relative to healthy individuals. This shift in BE suggests that a link might exist between the ratio of Gram-positive to Gram-negative taxa and EAC development.^13^^,^^14^ A hypothetical shift in Gram-positive to Gram-negative taxa ratio aligns well with the recently discovered competition model between two major bacterial groups or “guilds,” one guild of mainly Gram-positive bacterial taxa specialized in carbohydrate fermentation and production of the SCFA butyrate, and the other guild characterized by overrepresentation of mainly Gram-negative taxa associated with disease and antibiotic resistance that distinguish human disease from control cases.^15^ The relative increased abundance of disease-associated, often Gram-negative taxa observed in GERD may thus contribute to the inflammatory environment associated with BE and potentially influence its progression to EAC.

While acid suppression with proton pump inhibitors (PPIs) and endoscopic surveillance remain the standard of care for BE,^16^ these strategies do not reverse dysbiosis or restore microbial community function, and progression to neoplasia may still occur despite adequate therapy. Therefore, targeted modulation of the microbiome could represent a complementary therapeutic strategy, with probiotics offering a safe and non-invasive approach to restoring microbial balance and potentially positively affecting mucosal health in BE. Probiotic administration has shown promise in modulating gut microbiome composition in gastrointestinal conditions. While research on probiotics in the context of BE is limited, studies in related conditions show interesting insights. For instance, probiotic consumption of Gram-positive taxa, such as members of carbohydrate-degrading and SCFA-producing genera *Lactobacillus*, *Bifidobacterium*, and *Streptococcus*, may improve GERD symptoms.^17^ Probiotics may help restoring bacterial balance by increasing the abundance of Gram-positive species, potentially counteracting the shift toward Gram-negative, frequently disease-associated taxa and thus fitting the aforementioned “two-competing-guilds” model correlating with health or disease. *In vitro* studies using a BE model have demonstrated that probiotic consumption can be associated with substantially lower expression of key biomarkers associated with progression of BE to EAC. Of particular interest is *Lactobacillus casei* strain Shirota (LcS; new nomenclature: *Lacticaseibacillus paracasei* strain Shirota), frequently used in probiotic dairy products,^18^ which has demonstrated potent anti-tumor, anti-metastatic, and anti-proliferative effects in various cancer models, including gastrointestinal cancer.^19^^,^^20^^,^^21^^,^^22^ A component of the cell wall polysaccharide-peptidoglycan complex (PSPG) of LcS has been shown to counteract lipopolysaccharide (LPS)-induced IL-6 in the lamina propria of mice with murine IBD. Thus, in this model, Gram-positive LcS could counteract the effects of LPS from Gram-negative bacteria.^23^ Although the specific impact of probiotics on the esophageal microbiome in patients with BE requires further investigation, these preliminary findings indicate potential for manipulation of esophageal microbiota composition to improve BE management and EAC prevention.

We hypothesized that consumption of a probiotic LcS-containing beverage by patients with BE might result in an increase in the RA of Gram-positive taxa and a decrease in the RA of Gram-negative disease-associated taxa in the esophageal microbiome. To test this hypothesis, we invited patients with BE undergoing surveillance endoscopies to consume a probiotic drink containing LcS (Yakult) twice daily for 4 weeks. The goal of our study was to measure and monitor changes in bacterial composition following LcS-containing beverage consumption in MCE and NSE of patients with BE, mainly to investigate changes in the RA of Gram-positive and Gram-negative taxa.

## Results

### Patient enrollment and baseline characteristics

**Figure 1:**
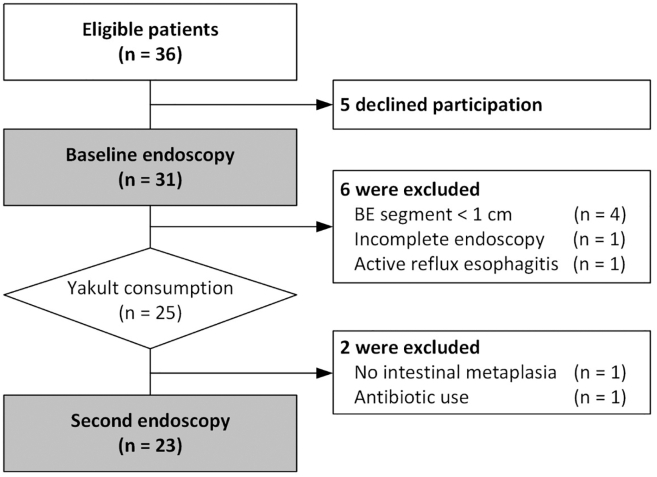
Study population

A total of 31 patients were enrolled and underwent baseline endoscopy. Of these, 25 patients who met inclusion criteria and had the necessary number of study biopsies taken started the 4-week Yakult intervention. Twenty-three patients completed the study, including the second endoscopy (Figure 1). Baseline characteristics are shown in Table 1. Analyses performed on patient samples are listed in Table S1. Most patients were male (73.9%), with a median age of 68 years (interquartile range [IQR] = 59–71). Median BE segment length was circumferential (C) 2 cm and maximum (M) 4 cm (IQR = C0–5, M3–7). All patients were on acid suppression therapy with either PPIs or H2 receptor antagonists, as this is standard clinical practice for BE. Patients had been on this therapy for at least one year prior to inclusion. Specifically, 22 of 23 participants received a once-daily dose of 40 mg of a PPI, while one participant used an H2 receptor antagonist due to intolerance to PPI therapy. No patients reported reflux symptoms at baseline, and endoscopic evaluation showed no signs of inflammation in any of the patients. Histopathological evaluation of clinical FFPE biopsies confirmed the presence of MCE with intestinal metaplasia in the BE segment without dysplastic changes in all patients at baseline.

**Table 1:** Patient characteristics

	*n* = 23	
**Clinical features**		

Gender, male (%)	17 (73.9%)	
Age (years), median (IQR)	68 (59–71)	

**Center, *n* (%)**		

Tertiary referral center	18 (78.3%)	
Secondary hospital	5 (21.7%)	
Body mass index (kg/m^2^), median (IQR)	27.2 (25.5–30.9)	
Smoking status—Ever smoked	12 (52.2%)	
Alcohol consumption—Current use	19 (82.8%)	
Special dietary pattern, *n* (%)	0 (0%)	
Proton pump inhibitor use, *n* (%)^a^	22 (95.7%)	

**Comorbidity, *n* (%)**		

Cardiovascular disease	3 (13.0%)	
Hypertension	4 (17.4%)	
Pulmonary disease	2 (8.7%)	
Chronic kidney disease	2 (8.7%)	
Previous malignancy	3 (13%)	
Diabetes mellitus	1 (4.4%)	

**Endoscopic features**		

Circumferential BE segment length (cm), median (IQR)	2 (0–5)	
Maximum BE segment length (cm), median (IQR)	4 (3–7)	
Hiatal hernia, *n* (%)	21 (91.3%)	

**Histologic features clinical biopsies (formalin-fixed)**		

Non-dysplastic BE	21 (91.3%)	
Low-grade dysplasia	2 (8.7%)	
	**Pre-LcS, *n* = 19**	**Post-LcS, *n* = 17**

**Histologic features study biopsies (methacarn-fixed)**		

Inflammation in BE biopsy		
No inflammation	7 (37%)	9 (53%)
Minimal chronic inflammation	9 (47%)	7 (41%)
Moderate chronic inflammation	3 (16%)	1 (5.9%)
Presence of MCE in BE biopsy	13 (68%)	10 (59%)
Presence of NSE in normal biopsy^b^	17 (94%)	16 (94%)

Inflammation and presence of metaplastic columnar epithelium (MCE) with intestinal metaplasia were scored in study biopsies only (Table S1). MCE was not present in 2 biopsies from BE only containing NSE (although clinical formalin biopsies confirmed MCE), 9 biopsies were taken unintentionally from or below gastroesophageal junctions. In 4 cases, methacarn-fixed biopsies were not available. In 2 additional cases, post-LcS tissue was not properly embedded resulting in inability to evaluate the tissue.

a 40 mg PPI once daily; 1 patient used an H2 receptor antagonist due to PPI intolerance.

b 1 additional NSE biopsy pre-LcS was not properly embedded.

### Clinical evaluation

Self-reported adherence to Yakult regimen was high, with 96% of patients reporting >90% compliance (1 diary missing). No serious adverse events were reported during the study period. Three mild adverse events were reported during the study: two cases of diarrhea and one case of bloating, all classified as grade 1 according to CTCAE v5.0 and not requiring any intervention. Following the 4-week Yakult intervention, the second endoscopy revealed no significant changes in endoscopic features or clinical characteristics compared to baseline. The absence of initial reflux symptoms or endoscopic inflammation made it not possible to evaluate any potential improvements due to Yakult consumption.

### Histopathological evaluation and *in situ* detection of bacteria

**Figure 2:**
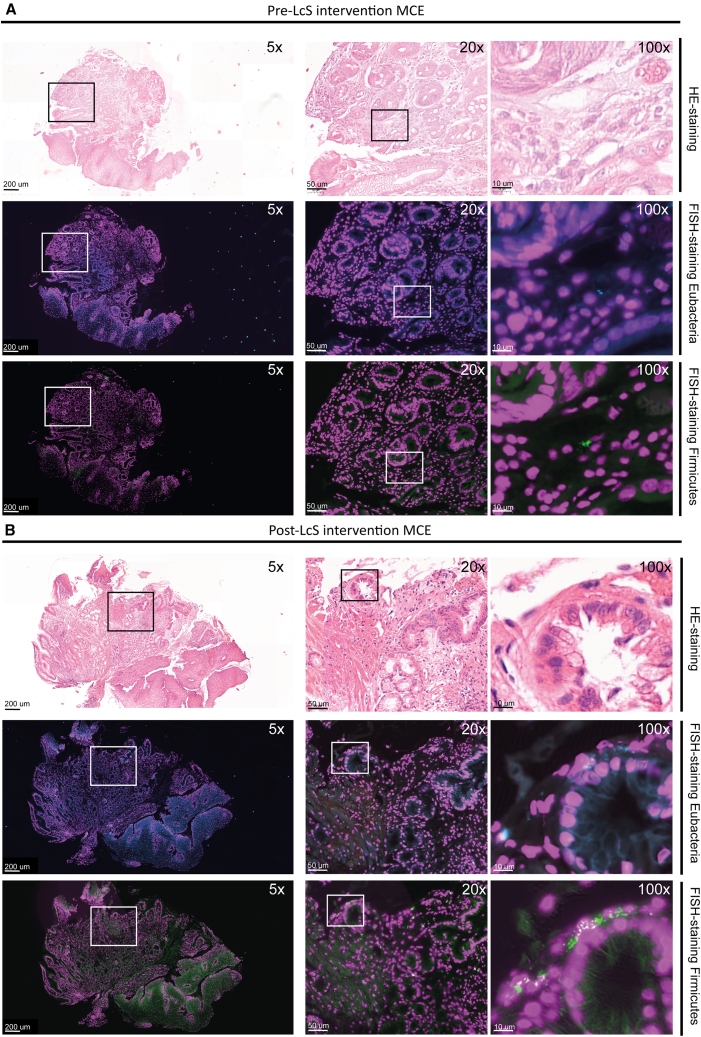
FISH detection of bacteria in MCE biopsies pre- and post-LcS-containing beverage consumption Representative H&E staining and corresponding FISH images of Eubacteria (cyan) and Firmicutes signals (green) detected in MCE biopsies of 1 case with high Eubacteria and Firmicutes presence pre-LcS-containing beverage consumption (A) and post-LcS-containing beverage consumption (B) at 5×, 20×, and 100× magnification. Scale bars are depicted. Nuclei are visible in magenta (DAPI stain).

Histopathological evaluation of the endoscopically guided Methacarn-fixed study biopsies, intended for microbial *in situ* bacterial evaluation, was scored for the presence of MCE and inflammation. In 68% and 59% of BE segment study biopsies, MCE with intestinal metaplasia was detected pre- and post-LcS-containing beverage consumption, respectively. Minimal or moderate chronic inflammation was observed in 63% and 47% of MCE biopsies pre- and post-LcS-containing beverage consumption, respectively (*p* > 0.05; Table 1). Histologic inflammation was not observed in any of the NSE biopsies. We evaluated the *in situ* presence of Eubacteria, Gram-positive Firmicutes (recently reclassified as Bacillota), and Gram-negative Bacteroidetes (recently reclassified as Bacteroidota) and Gammaproteobacteria using fluorescence microscopy. Representative images of histology, fluorescence *in situ* hybridization (FISH) for Eubacteria and Firmicutes are shown in Figure 2 for MCE, with corresponding controls and NSE biopsies in Figures S1 and S2. No differences were observed in the number of Eubacteria signals per mm apical region in both MCE (median 2.19 and 2.93) and NSE biopsies (median 1.35 and 0.96) pre- and post-LcS-containing beverage consumption, respectively. A significant increase in Firmicutes signals post-LcS-containing beverage consumption was observed per mm apical region for MCE biopsies (median 0.32 vs. 1.67; ∗∗*p* < 0.01, Wilcoxon signed-rank test). A similar (non-significant) trend was observed in NSE biopsies (median 0.4 vs. 0.86; Figure S3). The Gram-negative bacterial taxa Bacteroidetes and Gammaproteobacteria were detected in a minimal number of samples at low abundance pre- and post-LcS-containing beverage consumption.

### Gram-positive to Gram-negative ratio in MCE and NSE biopsies pre- and post-LcS-containing beverage consumption

Our working hypothesis was that the consumption of a Gram-positive LcS-containing probiotics drink (Yakult) would lead to an increase in the RA of Gram-positive taxa and a reduction in the RA of Gram-negative taxa. The Gram-positive to Gram-negative ratio measured with digital droplet PCR (ddPCR), a quantitative PCR method that counts DNA target copies in nanoliter-sized droplets, showed a higher RA of Gram-negative bacteria in both NSE and MCE regions pre-LcS compared with post-LcS-containing beverage consumption (Figure S4A). Post-LcS-containing beverage consumption, the Gram-positive to Gram-negative ratio had increased from 0.42 to 1.02 (∗∗∗*p* ≤ 0.005) in NSE regions and from 0.48 to 1.11 (*p* > 0.05) in MCE regions, covering 90% and 75% of the pairs, respectively (Figures S4B and S4C). We were unable to show specific colonization of the esophagus by the LcS strain in neither NSE nor MCE biopsies after twice daily consumption.

### General characteristics of esophageal microbiota in patients with BE at baseline

**Figure 3:**
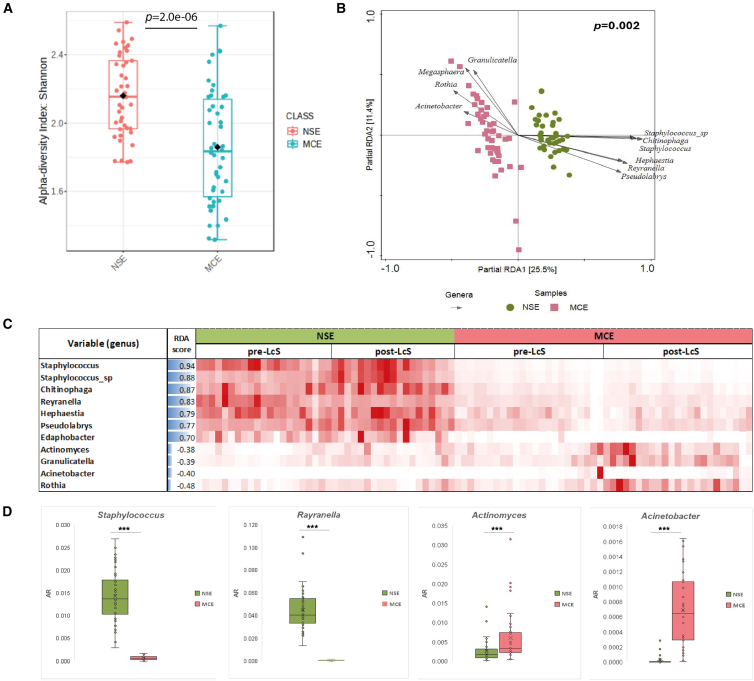
RDA and RA reveal the most discriminative genera in MCE vs. NSE (A) Differences in the Shannon α-diversity index of microbial communities at the genus level between esophageal locations (NSE and MCE). Boxplots indicate median ± IQR and min-max values. Diamonds represent the average. ∗∗∗*p* ≤ 0.005, Wilcoxon signed-rank test. (B) Partial redundancy analysis (RDA) of genera adjusted for patients and LcS-containing beverage consumption of esophageal region samples; the *x* axis separates NSE from MCE and explains 25.5% of the microbial composition differences observed; microbial community composition sampled from the two regions in the esophagus was significantly different (*p* = 0.002). The top 10 most discriminative genera are indicated with black arrows. The direction of arrows correlates with location, and arrow length correlates with correlation strength. (C) The relative abundance (RA) of the most discriminative genera displayed as heatmap. (D) Boxplots show RA of the most discriminative genera. Boxplots indicate median ± IQR and min-max values. Cross represents the average. ∗∗∗*p* ≤ 0.005, multiple linear regression adjusted with covariates: treatment and patients.

To assess whether the intake of an LcS-containing beverage could alter esophageal microbiota of patients with BE*,* we compared the microbial composition, alpha diversity, and RA between MCE and NSE biopsies. At the phylum level, patients with BE showed a high abundance of Gram-negative Proteobacteria (Pseudomonadota) (55.1%), followed by Gram-positive Firmicutes (Bacillota) (23.5%), Actinobacteria (Actinomycetota) (4.6%), and Bacteroidetes (Bacteroidota) (3.5%). The remaining taxa included Fusobacteria, Epsilonproteobacteria, and Patescibacteria. Among the Proteobacteria, the most common genus was *Mesorhizobium* (32.1%), followed by *Xanthobacteraceae* (12.2%%) (specifically *Bradyrhizobium*) and *Sphingomonas* (3.5%) (Figure S5A). Other common taxa included members of the Gram-positive Firmicutes such as *Streptococcus* (16.6%) and *Rothia* (Actinomycetota) (3.0%) and less abundant taxa such as the Gram-positive genus *Staphylococcus* (2.7%) and the Gram-negative genera *Prevotella* and *Reyranella* (both around 2%) (Figure S5B). The Shannon index, a standard measure of diversity that considers both the number of taxa and their proportional abundance, was significantly higher in NSE than in MCE at the genus level (*p* = 2.0e−06) (Figure 3A). To test the hypothesis that BE is associated with a higher RA of Gram-negative taxa, we compared the RA of the Gram-negative phylum Proteobacteria and the Gram-positive phylum Firmicutes between NSE and MCE biopsies. At the phylum level, no significant differences were observed between the RA of Proteobacteria (NSE = 66.3% vs. MCE = 62.8%) and Firmicutes (NSE = 26.0% vs. RA MCE = 25.5%) in MCE vs. NSE biopsies. In addition, we performed a partial redundancy analysis (partial-RDA) for tissue origin (MCE vs. NSE) and corrected this for the covariates LcS-containing beverage consumption and patients because these could at least partially explain variation in RA of specific microbiota taxa. The tissue origin MCE or NSE contributed 25.5% to the total variation in bacterial RA (*p* = 0.002), showing that the bacterial composition differed significantly between NSE and MCE of the esophagus (Figure 3B). The genera at highest differential RA in NSE included *Staphylococcus*, *Chitinophaga*, *Reyranella*, *Hephaestia*, and *Pseudolabrys* (RDA values = 0.70–0.94). In contrast, the genera *Rothia*, *Acinetobacter, Actinomyces*, and *Granulicatella* were at higher RA in MCE (RDA values = −0.48 to −0.38; Figures 3C and 3D).

### Higher bacterial diversity in BE and squamous epithelium post-LcS-containing beverage consumption

**Figure 4:**
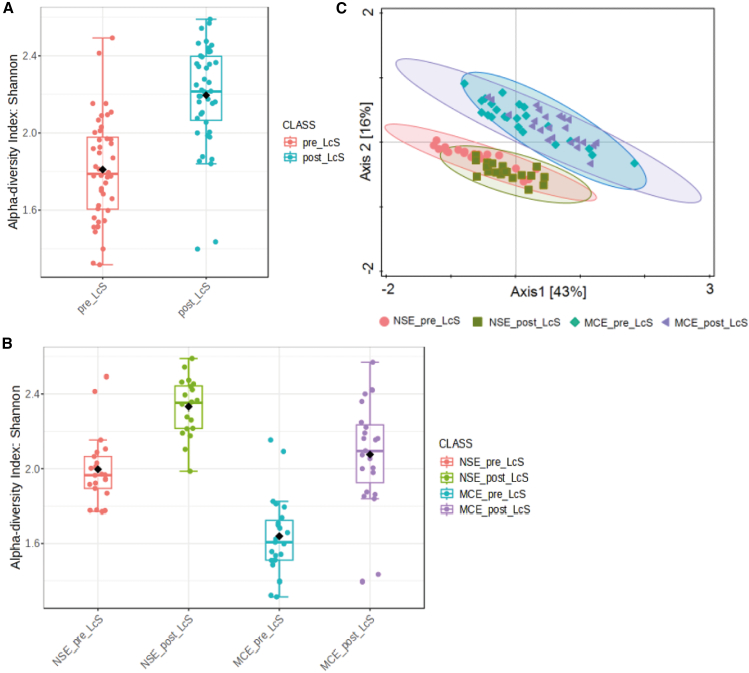
Differences in α- and β-diversity of MCE vs. NSE pre- and post-LcS-containing beverage consumption Differences in the Shannon α-diversity index of microbial communities at the genus level: (A) before and after intervention with LcS-containing beverage (pre-LcS and post-LcS). Boxplots indicate median ± IQR and min-max values. Diamonds represent the average. ∗∗∗*p <* 0.005, Mann-Whitney *U* test. (B) Pre-LcS and post-LcS for each esophageal region (both ∗∗∗*p* < 0.005). (C) Principal coordinate analysis (PCoA) visualizing differences in microbiota composition at the genus level associated with sampled esophageal regions and pre- or post-LcS-containing beverage consumption sampling time points. PCoA was generated using Bray-Curtis distance at the genus composition level. The experimental groups were distinct by esophageal tissue origin (NSE vs. MCE) and for intervention (pre- vs. post-LcS). ∗∗∗*p* < 0.005, PERMANOVA based on Bray-Curtis distance.

Next, we evaluated specific changes in bacterial diversity comparing pre- and post-LcS biopsies from NSE and MCE regions. Consumption of an LcS-containing beverage increased bacterial richness in both NSE and MCE esophageal locations, with significant differences observed at the genus level (*p* = 2.8e−05) (Figures 4A and 4B and Table S3). Multivariate permutational multivariate analysis of variance (PERMANOVA) based on Bray-Curtis distances revealed distinct microbial communities at the genus level that occurred at significantly differential RAs between NSE and MCE (*p* = 0.001) (Figure 4C), and between pre- and post-LcS-containing beverage consumption (Table S4). Principal-component analysis (PCA) corroborated these findings, showing that nearly 60% of the variation was captured by the first and second principal component (PC1&2) (Figure S6). Both the two esophageal regions (NSE vs. MCE) and the intervention (pre- vs. post-LcS) contributed statistically significant to the separation of microbiota composition (MCE vs. NSE, *p* = 0.001; pre-LcS vs. post-LcS, *p* = 0.001; Table S3). The genera that contributed most to the differences in microbiota composition between NSE and MCE regions included *Prevotella*, *Megasphaera*, and *Actinomyces*, the principal discriminating genera in MCE. In contrast, bacteria belonging to the genera *Staphylococcus*, *Reyranella*, and *Chitinophaga* were more dominant in NSE (Figure S6).

### LcS-containing beverage consumption increased specific Gram-positive taxa and reduced specific Gram-negative taxa

**Figure 5:**
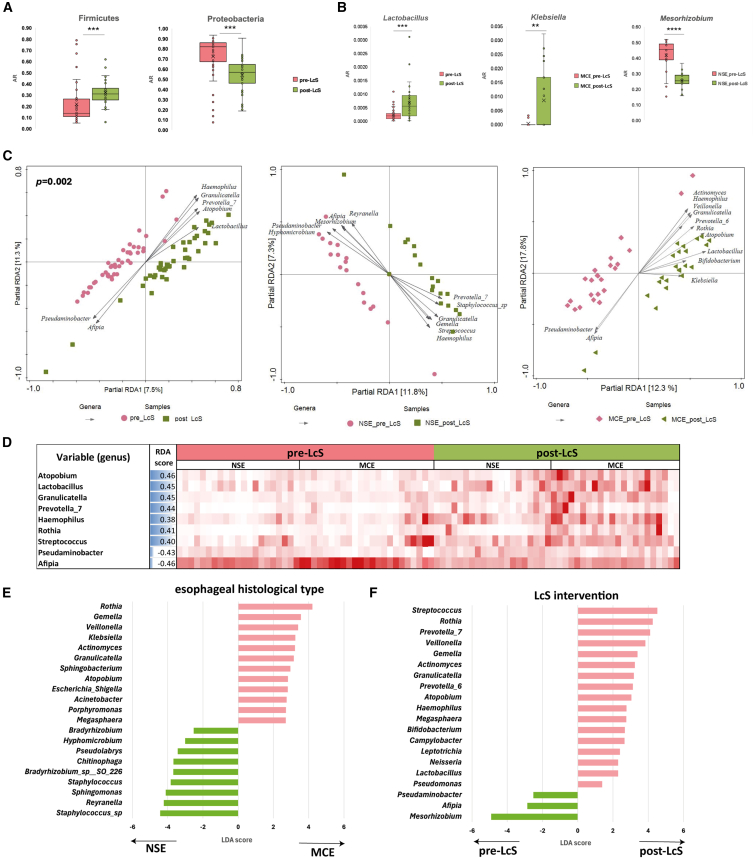
RDA analysis and RA identify the most discriminative genera pre- and post-LcS-containing beverage consumption (A) The relative abundance (RA) of the phyla Firmicutes and Proteobacteria before and after LcS-containing beverage consumption. ∗∗*p* < 0.01, ∗∗∗*p* < 0.005, ∗∗∗∗*p* < 0.001, Wilcoxon signed-rank test. (B) RA of the most discriminative genera *Lactobacillus* and *Mesorhizobium* in NSE and *Klebsiella* in MCE biopsies pre- vs. post-LcS-containing beverage consumption. ∗∗*p* < 0.01, ∗∗∗*p* < 0.005, ∗∗∗∗*p* < 0.001, Wilcoxon signed-rank test. Boxplots indicate median ± IQR and min-max values. Cross represents the average. (C) Partial-RDA of genera grouped for NSE and MCE together comparing pre- with post-LcS tissue of origin (MCE vs. NSE) and intervention (pre- vs. post-LcS) separately. The RDA1 axis separates pre-LcS from post-LcS and explains 6.5% of observed differences in bacterial composition. Bacterial community composition changed significantly due to consumption of LcS-containing beverage (*p* = 0.002). The top 10 most distinctive genera are represented with black arrows. Arrow directions correlate with esophageal location, and arrow lengths correlate with correlation strength. (D) RA of the most discriminative genera displayed as heatmap. (E and F) (E) Rank correlation analysis using linear discriminant analysis (LDA) effect size (LEfSe) was performed to compare microbial features, candidate biomarkers, at the genus level between esophageal regions (NSE and MCE) and (F) before and after consumption of LcS-containing beverage. Phylum with LEfSe scores >2.5 or <−2.5 and an FDR-corrected *p* < 0.05 were considered significant. ∗∗*p* ≤ 0.01, ∗∗∗*p* ≤ 0.005, multiple linear regression adjusted with covariates: esophageal regions and patients.

Consumption of an LcS-containing beverage led to a significant increase in the RA of Gram-positive Firmicutes at the phylum level, showing an RA increase from 22% to 32% in both NSE and MCE biopsies (Tukey’s Honestly Significant Difference (HSD) test: ∗∗∗*p* ≤ 0.005, *p* < 0.05, respectively). Gram-negative Proteobacteria showed a reduction in RA from 72.0% to 54% (Tukey’s HSD: ∗∗*p* ≤ 0.01 for both NSE and MCE) (Figures 5A and S4C). Among Firmicutes, the RA of the genus *Lactobacillus* had increased from 0.01% to 0.03% (Tukey’s HSD: ∗∗∗*p* ≤ 0.005 for MCE only) (Figure 5B). The significant increase in Firmicutes and reduction in Proteobacteria was observed only in patients included at the Radboudumc hospital and not at the Bernhoven site (Figure S7).

The impact of LcS-containing beverage consumption on microbiota composition at the genus level was assessed using partial-RDA, adjusted for patients and tissue origin (MCE vs. NSE phenotype; detailed Methods in supplemental information; Figures 5C and 5D). Partial-RDA revealed that the intervention (LcS beverage consumption) explained 7.5% variation in microbiota composition of the esophageal microbiota pre- and post-LcS. Post-LcS-containing beverage consumption, genera that contributed strongest to differences in microbiota composition in the esophageal tissue origin included *Lactobacillus*, *Rothia*, *Streptococcus*, *Veillonella*, and *Klebsiella* (Figures 5C and 5D). The partial-RDA for NSE and MCE showed shared genera significantly contributing to the variation, such as *Haemophilus* and *Prevotella*, but also specific contributions for MCE alone, such as *Lactobacillus, Bifidobacterium*, and *Klebsiella.*

We also investigated which genera had contributed most to the differential microbiota composition in the NSE and MCE regions. In the NSE region, LcS-containing beverage consumption led to a significant reduction in Gram-negative *Mesorhizobium*, from 41% to 25% (∗∗∗∗*p* ≤ 0.001) (Figure 5B). Although LcS-containing beverage consumption led to a decrease in Gram-negative Proteobacteria, it also resulted in an increase in the taxa *Prevotella* from 0.8% to 2.9% in NSE and 2.9% to 4.4% in MCE regions. Members of the genus *Haemophilus* and *Klebsiella* also showed a significant increase in MCE biopsies (∗∗*p* < 0.01), but remained at very low RA (0.1%–0.5%) (Figures 5B and S7). Next, we used linear discriminant analysis effect size (LEfSe), specifically designed for biomarker discovery rather than multivariate analysis of RA, across sampling regions. LEfSe determines the taxa most likely to explain differences in microbiota composition between NSE and MCE by coupling standard tests for statistical significance with additional linear discriminant analysis (LDA) tests to improve biological consistency and impose a given effect size.^24^ We used LEfSe to identify taxa associated with BE by comparing microbiota composition between NSE and MCE biopsies and to identify (biomarker) taxa associated with impact of LcS-containing beverage consumption. LEfSe analysis corroborated our previous findings, uncovering Firmicutes as a biomarker post-LcS-containing beverage consumption (LDA score = 4.66, *p* ≤ 0.005; Table S4). Members of the phyla Proteobacteria (Pseudomonadota) and Fusobacteria (Fusobacteriota) were listed as enriched candidate biomarkers pre-LcS-containing beverage consumption (Figure S6 and Table S5). According to LEfSe analysis, members of the genera *Staphylococcus*, *Reyranella*, and *Chitinophaga* were biomarkers associated with NSE regions, whereas members of the genera *Streptococcus, Rothia*, and *Prevotella_7* were determined as post-LcS-containing beverage biomarkers (Figures 5E and 5F).

## Discussion

Our study highlights significant differences in bacterial composition of NSE and MCE in patients with BE, accounting for 25.5% of the observed variation. Consumption of the LcS-containing beverage Yakult led to notable shifts in the esophageal microbiota composition, including increased microbial diversity in both NSE and MCE epithelial regions and a significant increase in the Gram-positive/Gram-negative ratio. Specifically, we observed a marked increase in the RA of Gram-positive Firmicutes (from 22% to 32%) alongside a reduction in Gram-negative Proteobacteria (from 72% to 54%). The most significantly increased Gram-positive taxa post-LcS-containing beverage consumption was *Streptococcus*, increasing from 12% to 17% in NSE and 18% to 24% in MCE, whereas *Lactobacillus* RA increased significantly but only marginally from 0.01% to 0.03%. Some taxa previously associated with BE and EAC, such as *Prevotella*,^25^ also showed increased RA post-LcS-containing beverage consumption in both epithelial regions (from 0.08% to 2.9% in NSE and 2.9% to 4.4% in MCE). These findings suggest that although LcS-containing beverage modulates the esophageal microbiota toward a more diverse and higher Gram-positive RA composition, undesired increases in RA of disease-associated taxa, such as *Prevotella*, warrant further investigation.

Our finding that within the same individual, esophageal squamous and metaplastic epithelium exhibit substantially different bacterial compositions as a consequence of LcS-containing beverage consumption underscores the importance of the local epithelial environment in shaping the esophageal microbiome. Higher bacterial diversity, especially of Gram-positive taxa, were features of a healthy esophageal squamous epithelium, whereas Gram-negative bacteria appeared to be abundant in metaplastic epithelium, associated with lower bacterial diversity. This observed Gram-negative predominance in BE is of clinical relevance. The outer membrane of Gram-negative bacteria possesses LPS, potent inducers of inflammation, that may contribute to the metaplasia-dysplasia-carcinoma sequence observed in BE.^13^ Furthermore, certain Gram-negative species found in BE, such as *Campylobacter concisus*, produce toxins that damage epithelial cells and potentially contribute to carcinogenesis.^26^^,^^27^ More generally, progression from healthy NSE in the esophagus to BE-associated MCE and subsequently to EAC is known to be characterized by an increased RA of Gram-negative bacterial species,^13^ including disease-associated species from the genera *Campylobacter* and *Fusobacterium*.^28^^,^^29^^,^^30^ Conversely, Gram-positive bacteria, particularly certain *Streptococcus* and *Lactobacillus* species, of which we assayed the strain *L. casei* Shirota, may play protective roles in esophagus through production of bacteriocins, SCFAs from the breakdown of complex carbohydrates, and competition with pathogenic species.^8^ The SCFAs lactate and acetate modulate the immune response, host tolerance, and contribute to competitive capacity of Gram-positive bacteria against pathogenic, often Gram-negative, microorganisms.^31^^,^^32^ The contrasting ecological properties of Gram-negative and Gram-positive taxa align with the recently discovered model of two competing guilds, one mainly containing Gram-negative taxa that include common disease-associated species and lineages, and another one containing Gram-positive taxa associated with anaerobic fermentation, butyrate production, and health.^15^ Interestingly, we found that Gram-negative *Mesorhizobium*, overrepresented in patients with GERD^33^^,^^34^ and at high RA in squamous epithelium of our patients, had significantly decreased after 4 weeks of LcS-containing beverage consumption. Because no initial endoscopic inflammation was seen in any of the subjects, we could not evaluate the effect of LcS-containing beverage consumption and the corresponding microbiome shift on endoscopic inflammation. Histologic mild inflammation was present in 63% of patients before LcS-containing beverage consumption and had decreased to 46.9% after LcS-containing beverage consumption, although not statistically significant, which may suggest that a longer intervention period and larger sample size could potentially allow a more persistent increase in Gram-positive RA and reduction in histological inflammation.

While our study focused on the probiotic fermented drink Yakult containing high amounts of LcS, the matrix of the drink could also have contributed to the observed effects. In the current design, we are unable to discriminate whether the observed microbiota changes are due to LcS or any other components in the matrix, such as sugar or milk components. In the metaplastic esophageal region, we observed an increased RA of typical intestinal taxa such as *Prevotella*, which could be driven by gastroesophageal reflux. However, also the high glucose content in the probiotic Yakult drink may have stimulated growth of pro-inflammatory bacteria and decreased capacity of mucosal tissues to regulate epithelial integrity and immunity. Higher RA of *Prevotella,* a taxon associated with BE, dysbiosis, and cancer,^30^^,^^35^ warrants also further attention, as previous studies have linked *Prevotella* to inflammatory conditions and tissue damage through the production of pro-inflammatory mediators^36^^,^^37^ that may drive inflammation-driven oxidative stress. Importantly, histologic examination of inflammation post-LcS-containing beverage consumption showed no significant increase or reduction of inflammation. Longer intervention periods may be necessary to study the net effect of the microbiota shift on inflammatory parameters and to rule out any negative effects of the intervention.

Comparable to the potential effects of glucose on *Prevotella* proliferation*,* homolactic fermentation of glucose by LcS and other Gram-positive bacteria, such as *Streptococcus*, potentially have a dual effect: modulating host immunity via lactate and acetate, and decreasing pH.^38^^,^^39^ The RA of *Streptococcus* and *Rothia* post-LcS-containing beverage consumption contributed strongly to the significant increase in Firmicutes, while the RA of the genus *Lactobacillus* remained low (0.01%–0.03%). In this study, we observed no colonization with LcS using an LcS-specific assay, suggesting that other effects of the LcS or the beverage matrix, such as changing community dynamics via production of lactate and acetate, may be the primary mechanism of action. To optimize investigating any probiotic effect of LcS-containing beverages as an intervention to improve BE, a carefully designed placebo-controlled trial with LcS matrix alone, an increased number of subjects for better power, and a longer follow-up period after intervention are necessary to evaluate important clinical parameters and assess supplementation potential. It is advisable to design a probiotic formulation with low or no glucose content and combine this together with acid-inhibiting agents such as PPIs, which might enhance probiotic benefits, including reducing the RAs of disease-associated Gram-negative bacteria.

The findings of our study highlight differences in the esophageal microbiome in BE at baseline (before intervention). First, microbial composition within metaplastic Barrett’s epithelium may serve as a biomarker to identify patients at higher risk of progression to dysplasia or EAC. Specifically, increased abundance of taxa such as Proteobacteria, Fusobacteria, and *Prevotella* has been associated with inflammation and dysbiosis, possibly indicative of disease progression. Higher abundance of *Leptotrichia* (phylum Fusobacteria) was proposed by others as a candidate biomarker of neoplastic esophageal mucosa.^25^ Changes in microbiota composition contributing to, or resulting from, carcinogenic processes have positioned microbiome composition, especially RAs of Gram-positive and Gram-negative taxa, as biomarker for disease progression and therapeutic targets.^40^ To date, no studies have yet tried to test or implement any of these biomarkers to monitor disease progression, which is a next key step to test the feasibility of such an approach.

The observed effects of Yakult intervention on microbiome diversity, especially the Gram-positive/Gram-negative ratio, suggest the potential of manipulating microbiota composition in BE. However, therapeutic benefit could not be assessed in this study, as the study setup did not allow distinguishing between the effect of the beverage matrix and LcS. Biological mechanisms of LcS consumption should be explored in prolonged and placebo-controlled studies that also take the beverage matrix into account. In a previous study, the *Streptococcus* (Gram-positive) to *Prevotella* (Gram-negative) ratio was negatively associated with risk factors for BE pathogenesis, such as the waist-to-hip ratio, suggesting that reverting the *Streptococcus* to *Prevotella* ratio could be beneficial.^41^ By enhancing microbial diversity and improving the balance between Gram-positives and Gram-negatives, future probiotic interventions, together with dietary advise and medication where appropriate, could mitigate inflammation and support epithelial integrity, thereby reducing EAC risk. Results from a BE organoid model support potential biological relevance, as L-lactate production by *Streptococcus* and *Lactobacillus* reduced EAC proliferation and modulated immune and inflammatory responses.^38^ Therapeutic modulation of the esophageal microbiota, through Gram-positives such as LcS or other targeted interventions that stimulate an increase in Gram-positive taxa, thus represents a promising avenue to explore for BE management, complementing current medical and endoscopic management aimed at halting or reversing disease progression.^42^

This study included a thorough microbiome analysis using 16S rRNA sequencing, ddPCR, and FISH, providing a comprehensive view of the esophageal microbiota in patients with BE. Studying squamous and metaplastic epithelium within the same subjects offered valuable insights into personalized, region-specific microbial differences. Furthermore, the within-subject design allowed for robust assessment of changes induced by LcS-containing beverage consumption. However, limitations should be considered when interpreting the results (see limitations of the study).

In conclusion, LcS-containing beverage consumption in patients with BE led to an increase in bacterial diversity and RA of Gram-positive taxa, together with a decrease in RA of Gram-negative taxa. Although remaining at low RA, the concurrent relative increase in specific disease-associated taxa that thrive on simple sugars, such as *Prevotella*, *Haemophilus*, and *Campylobacter*, suggests that careful formulation of probiotic drinks is desired. Our findings underscore the potential of microbiome modulation through probiotic formulations in a relatively short intervention period and offer leads for placebo-controlled trials with clinical read-outs to evaluate a possible therapeutic strategy in BE management and EAC prevention.

### Limitations of the study

The relatively small sample size may limit generalizability of the findings. We noted differences in microbiome composition between patients included in different hospitals, underscoring that a more diverse study population is necessary to generalize conclusions. The intervention duration of 4 weeks, while sufficient to observe some changes, may not fully capture long-term effects of probiotic consumption on the esophageal microbiome. In addition, the lack of a control group without probiotic consumption, and/or a group that consumed the Yakult milk matrix but without viable LcS bacteria, makes it challenging to distinguish between LcS intervention effects and potential confounding factors such as the nutrient medium (the milk matrix of the formulation). We acknowledge that it would be of interest to design future studies that also take natural fluctuations of microbiota composition over time into account. For this exploratory, proof-of-concept study, we chose a single-arm design to investigate if a shot-term intervention could induce measurable microbiome shifts. The present findings should therefore be viewed as mainly hypothesis generating that may form the basis for a future randomized controlled trial with a carefully designed probiotic LcS formulation. Future research directions could address these limitations and expand our understanding of the esophageal microbiome in BE, including mechanistic studies to elucidate the underlying processes driving microbial shifts in BE and their potential role in disease progression.

## Resource availability

### Lead contact

Further information and requests for resources and reagents should be directed to and will be fulfilled by the lead contact Dr. Annemarie Boleij (annemarie.boleij@radboudumc.nl).

### Materials availability

This study did not generate new unique reagents. The probiotic product (Yakult, containing *Lactobacillus casei* strain Shirotais commercially available. Biopsy material embedded in Methacarn or leftover DNA is available upon request and can be shared with an MTA with GDPR compliance.

### Data and code availability

• The raw 16S rRNA gene sequencing data generated in this study have been deposited in the European Nucleotide Archive (ENA) under accession number PRJEB89339. The processed relative abundance data are available in Table S6.
• No custom code was generated, all the tools used for sequence processing are available in the respective methods section.
• Any additional information required to reanalyze the data reported in this paper is available from the lead contact upon request.

## Acknowledgments

The study was partly funded by Yakult Europe B.V. for an amount of 40.000 euro. Yakult Europe B.V. did not have any input in the design, execution, or interpretation of the outcome of the study.

## Author contributions

Conceptualization, P.D.S., Y.P., and P.v.B.; data curation, Y.P., M.L.F., and A.B.; formal analysis, Y.P., M.L.F., C.Z., R.t.M., B.v.d.L., R.C., P.D.L., L.v.D., P.v.B., R.S.v.d.P., P.D.S., and A.B.; funding acquisition, P.D.S.; investigation, Y.P., R.W.M.S., A.C.T., and P.D.S.; methodology, Y.P., M.L.F., R.t.M., R.C., P.D.L., P.v.B., P.D.S., and A.B.; project administration, Y.P. and A.B.; resources, P.D.S., P.v.B., and A.B.; software, M.L.F. and P.v.B.; supervision, P.D.S., A.B., and P.v.B.; validation, Y.P., M.L.F., P.D.S., P.v.B., and A.B.; visualization, Y.P., M.L.F., C.Z., L.v.D., P.v.B., and A.B.; writing – original draft, Y.P., M.L.F., P.v.B., P.D.S., and A.B.; writing – review and editing, all authors.

## Declaration of interests

P.D.S. received unrestricted grants from PENTAX, FUJIFILM, Norgine, Micro-Tech, Magentiq Eye, AstraZeneca, Sanofi, and in the advisory board of Sanofi (The Netherlands). The study was partly funded by Yakult Europe B.V. for an amount of 40.000 euro. Yakult Europe B.V. did not have any input in the design, execution, or interpretation of the outcome of the study.

## STAR★Methods

### Key resources table

**Table:** 

REAGENT or RESOURCE	SOURCE	IDENTIFIER
**Bacterial and virus strains**		

Yakult fermented probiotic drink	Yakult Nederland BV, Amstelveen, The Netherlands	NA

**Chemicals, peptides, and recombinant proteins**		

DAPI	Thermo Fisher Scientific	P36931
Proteinase K	Qiagen	19133
Saponin	Sigma Aldrich	SAE0073
TurboDNase	Thermo Scientific	AM2238
mutanolysin from *Streptomyces globysporus* (ATCC 21553)	Sigma Aldrich	M9901
DNEasy powerlyser powersoil DNA isolation kit	Qiagen	12855
ddPCR Mastermix	Biorad	1864037

**Deposited data**		

Raw 16s rRNA amplicon sequencing data	European nucleotide archive	Accession no PRJEB89339
Trial protocol	Netherlands Trial Register	NL-OMON43164

**Oligonucleotides**		

See Table S2 for FISH probes	N/A	N/A
5′-TACGGGAGGCAGCAGT-3′	Primer PLK1	Wouters et al.,^43^ Klaschik et al.^44^
5′-TATTACCGCGGCTGCT-3′	Primer PLK2	Wouters et al.,^43^ Klaschik et al.^44^
5′-*FAM-*CTAACCAGAAAGCC ACGGCTAACTACGTG–*OQA*-3′	Ineternal hybridization probe Gram-positive	Wouters et al.,^43^ Klaschik et al.^44^
5′-*HEX*–TTACCCGCAGAATA AGCACCGGCTAAC–*BHQ1*-3′	Internal hybridization probe Gram-	Wouters et al.,^43^ Klaschik et al.^44^
5′-CTCAAAGCCGTGACGGTC-3′	Primer pLcS-57F	Fujimoto et al.^45^
5′-CACTAGGATTATTAGCACCACGT-3′	Primer pLcS-597R	Fujimoto et al.^45^
5′-*FAM*-CCTCTTGGGG AACCAGTGCAGCAG-3′	Internal hybridization prove LcS	designed in this study

**Software and algorithms**		

Quantasoft Software	BioRad	version 1.7.4
GraphPad Prism	Dotmatics	10 version 1.2
Canoco	Microcomputer Power	Version 5.10
FLASH	Johns Hopkins Univeristy/Center for computational biology	Version 1.2.7
QIIME	QIIME.org	Version 1.7.0
Uparse	Drive5.com	Version 7.0.1001
Mothur	Mother.org	Version 1.48.1
MUSCLE	Drive5.com	Version 3.8.31
R software	R foundation for statistical computing	Version 2.15.3

### Experimental model and study participant details

#### Human subjects

This single-arm, interventional pilot study included patients with Barrett’s esophagus (BE) undergoing surveillance endoscopy at Bernhoven Hospital (Uden, the Netherlands) and Radboud university medical center (Nijmegen, the Netherlands). Eligible participants were ≥18 years of age with a Barrett segment ≥2 cm and histologically confirmed intestinal metaplasia without dysplasia. Study participants who had been referred for a surveillance upper endoscopy for BE were recruited. Exclusion criteria included probiotic or antibiotic use within the last 3 months before baseline, infection of the oral cavity, a vegetarian or gluten-free diet, lactose intolerance, immunodeficiency disorders, bleeding disorders, *Helicobacter pylori* infection, previous gastric or esophageal surgery, and other coexistent esophageal diseases (e.g., varices or reflux esophagitis). The study was in accordance with the Declaration of Helsinki, the code of conduct for Health Research, and was approved by ethics committee CMO Arnhem Nijmegen (NL59072.091.16). All the participants provided informed written consent. All authors had access to the study data and reviewed and approved the final manuscript. A total 31 patients were asked to participate of which 23 participants completed the study. The mean age was 68 (IQR 59–71), and 73,9% of participants were male. All participants had BE with a median Prague classification of Circumferential extent 2 (C2), and Maximal Extent of 4 (M4). All participant used proton pump inhibitors (PPIs).

### Method details

#### Study design

We performed a single-arm, interventional pilot study to evaluate the ability of changing the esophageal microbiota by ingestion of a fermented milk drink containing LcS (Yakult) in patients will be with confirmed MCE with intestinal metaplasia. We analyzed the microbial composition of biopsies obtained from NSE and BE regions (MCE) before and after consumption with the LcS-containing beverage to identify potential alterations in mucosal microbiota composition. In addition, the ability of LcS to colonize the esophagus and the impact of the drink on the Gram-positive to Gram-negative ratio were investigated by 16s rRNA DNA sequence profiling of bacteria and fluorescent *in situ* microscopy analysis of biopsy sections.

#### Primary outcomes

The primary outcomes were (i) the effect of LcS-containing beverage on the Gram-positive to Gram-negative ratio in the esophagus, and (ii) the effect on the diversity and composition of the microbiota in NSE and MCE epithelium. Secondary outcomes were the impact of LcS-containing beverage on endoscopic and histologic inflammation.

#### Intervention

Patients received a fermented probiotic drink (Yakult: 65 mL; Yakult Nederland BV, Amstelveen, the Netherlands) containing a minimum of 6.5·10^9^ colony-forming units (CFU) LcS/bottle twice-daily for four weeks. Other Yakult ingredients included water, skimmed milk powder, glucose/fructose syrup, sugar (total added sugar 8.8 gr/100 mL), maltodextrin, and flavorings. We chose to use this probiotic LcS-containing beverage since this drink is generally regarded as safe, with clinical validation.^46^^,^^47^^,^^48^ Fourteen fresh bottles of Yakult were delivered by a courier service to the participants’ home addresses every week. Patients were asked to fast 15 min before and after drinking the bottle. Participants were instructed to take their final dose the evening before the follow-up endoscopy, as they were fasting on the day of the procedure. Compliance was assessed by a patient diary and registration of the remaining unopened bottles by the courier service. Adverse events were graded according to the Common Terminology Criteria for Adverse Events (CTCAE) version 5.0 (National Cancer Institute, U.S. Department of Health and Human Services). During the intervention period, patients were not allowed to take other probiotics or antibiotics.

#### Sampling procedures

At baseline and after 28 ± 3 days of LcS intake, participants underwent upper endoscopy with biopsy sampling. All study endoscopies were performed by one experienced endoscopist (PDS). The length of BE was measured using the Prague classification.^49^ Only patients without visible signs of inflammation (e.g., erosions, ulceration, or erythema) were included. At both endoscopies, four-quadrant biopsies were taken every 2 cm according to the Seattle protocol, with additional targeted biopsy of any macroscopic abnormalities.^50^ Biopsies were fixed in buffered formalin for histopathological examination by an experienced gastrointestinal pathologist.

Additional biopsy samples from NSE (*n* = 4) and MCE (*n* = 4) were taken for study purposes during each endoscopy. To minimize cross-contamination, sterile-forceps were used and samples of MCE were collected first before NSE. The biopsy forceps were washed in sterile water between sampling to remove possible tissue remnants between biopsies. MCE biopsies were obtained at least 1 cm above the gastroesophageal junction and biopsies of NSE at least 3 cm above the squamocolumnar junction. For each patient, 2 NSE and 2 MCE biopsies were fixed in Guanidine Hydrochloride for DNA and RNA isolation of LcS-analysis and microbial qPCRs and 1 NSE and 1 MCE biopsy were fresh frozen in liquid nitrogen and immediately transferred to −80°C for 16s rRNA gene analysis of the esophageal microbiome. Final study biopsies (1 NSE and 1 MCE) were fixated in Methacarn (30% chloroform, 10% acetic acid, 60% methanol) for *in situ* bacterial analysis. Study biopsies were evaluated by a trained pathologist for the presence of BE (metaplastic epithelium with intestinal metaplasia), gastric esophageal junction, gastric cardia or squamous epithelium in HE stained sections. An inflammation score from no, minimal, moderate or severe chronic inflammation was assigned. Researchers who performed subsequent processes were blinded to clinical information. For analysis performed on each biopsy see Table S1.

#### Histology and FISH

Tissue sections of 4 μm were used for Hematoxylin/Eosin (HE) staining and FISH. Stained slides were scanned at 20× magnification using the Pannoramic 1000 scanner for HE slides and Pannoramic Midi fluorescence scanner (3D Histech) for FISH with the following exposure times: DAPI 20 ms, FITC 40 ms, TRITC 38 ms, and Cy5 38 ms. HE slides were scored by a pathologist to verify presence of NSE and MCE in the obtained biopsies from each location. FISH was performed to detect bacteria in NSE and MCE tissue samples. Tissue sections were deparaffinized in xylene and rehydrated through a series of ethanol washes. FISH probes (EUB338-cy5 and non-EUB338-cy5) were diluted in hybridization buffer (0.9 M NaCl, 20 mM Tris-HCl pH 8.0, 0.01% sodium dodecyl sulfate) and added to airdried sections. For the probe mixture to detect *Bacteroidetes* (Alexa 488), Firmicutes (cy3), and Gammaproteobacteria (cy5), 20% formamide was added to the hybridization buffer to optimize hybridization of the probes. All probes were tested on protoblocks of the bacterial strains *Streptococcus gallolyticus, Escherichia coli* and *Bacteroides fragilis,* and colon epithelial tissue containing a biofilm to test specificity of the probes (Figure S1). Sections were incubated in humidified box overnight at 46°C and washed 3 times for 5 min in a washing buffer at 48°C (0.9M NaCl, 20 mM Tris-Cl pH 8.0), followed by 1 min iced H_2_O and 1 min in PBS. Slide mounting was performed with Prolong Gold Antifade medium with DAPI (Thermo Fisher Scientific, P36931). The EUB338 probe was used for all slides and for the positive control and the non-EUB probe to verify positive signals and exclude unspecific probe binding. FISH was carried out with oligonucleotide probes targeting bacterial 16S rRNA as listed in Table S2.

#### Scoring of HE and FISH slides

Each biopsy from NSE and MCE regions was evaluated by a pathologist specialized in upper gastointestinal pathology (R.S. van der Post) to confirm that NSE biopsies were from squamous epithelium and to confirm that the MCE regions contained columnar epithelium representative of BE. Each FISH image was compared to a non-EUB stained control to determine specificity of the signals. First the length of the apical region in the biopsy was determined to control for the size of the biopsies represented in the slices that were taken. Next all positive signals along the length of the epithelium were counted and divided by the length of the epithelium to generate number of bacteria per mm tissue. The same method was perform for each probe (LGC-mix, CFB, Gam42 and Eub338). All FISH slides were scored by two observers and a third for confirmation of the results.

#### DNA isolation

Endoscopic biopsies collected in liquid nitrogen were utilized to extract DNA from bacteria present in MCE and NSE. DNA extraction from mucosal biopsies was performed according to a previously optimized protocol.^51^ Briefly, frozen biopsies were placed in PBS, thawed and mixed for 5 min. Loosely attached bacteria were collected by removing the PBS from the biopsy. This biopsy wash was stored in a sterile Eppendorf tube on ice for later extraction. Firmly attached bacteria were recovered by proteinase K (Qiagen 19133) tissue digestion. The digested biopsy and PBS wash were combined; human DNA was digested by a combination of mild lysis with 0.0125% saponin (preserving bacterial cells) and DNAse digestion with 2U/μL (TurboDNAse AM2238, Thermo Fisher Scientific). Intact bacteria were collected by centrifugation at 10,000g for 10 min at 4°C. The bacterial pellet was suspended in Power bead solution containing 0.5U/μL mutanolysin from *Streptomyces globisporus* (ATCC 21553) and incubated at 37°C for 60 min. Subsequent DNA extraction was performed according to the DNeasy Powerlyzer Powersoil kit (Qiagen 12855), including bacterial lyses with 0.1 mm glass beads using the Fisherbrand Bead Mill 4, two times at maximum speed for 30 s with 30 s rest on ice in between. The total DNA yield was quantified using the Qubit 5.0 Fluorometer Broad Range DNA assay (Cat #Q32853, Life Technologies, USA), following the manufacturer’s guidelines. DNA quality was assessed by DNA electrophoresis in 1% agarose.

#### Quantitative PCR analysis for Gram-positive/Gram-negative ratio and LcS

To determine the ratio of Gram-positive (G+) and Gram-negative (G-) bacteria one set of primers and two probes were used. Primers PLK1 (5′-TACGGGAGGCAGCAGT-3′) and PLK2 (5′-TATTACCGCGGCTGCT-3′) targeting bacterial 16S rRNA were selected for amplification.^43^ Internal hybridization probes G+ (5′-*FAM-*CTAACCAGAAAGCCACGGCTAACTACGTG–*OQA*-3′) and G−(5′-*HEX*–TTACCCGCAGAATAAGCACCGGCTAAC–*BHQ1*-3′) were used for detecting the amplified template.^44^ For *Lactobacillus casei* strain Shirota (*LcS)* detection, the *LcS-*specific primer set pLcS with primers pLcS-57F (5′-CTCAAAGCCGTGACGGTC-3′) and pLcS-597R (5′-CACTAGGATTATTAGCACCACGT-3′) were selected for amplification.^45^ The internal hybridization probe LcS (5′-*FAM*-CCTCTTGGGGAACCAGTGCAGCAG-3′) was designed to detect the amplified DNA template of the pLcS primer set. The primer-probe combination was validated using LcS DNA and a set of pooled DNA from 17 different bacteria as negative control (*Acinetobacter baumannii, Enterococcus faecalis, Enterococcus faecium, Citrobacter freundii, Enterobacter cloacae, Staphylococcus aureus, Escherichia coli, Staphylococcus epidermidis, Klebsiella oxytoca, Klebsiella pneumoniae, Streptococcus agalactiae, Streptococcus pneumoniae, Streptococcus pyogenes, Proteus mirabilis, Morganella morganii, Pseudomonas aeruginosa, Staphylococcus warneri)*. The qPCR reactions were performed in duplicate using the QX200 Droplet Digital PCR system (Bio-Rad, CA, USA) in a final volume of 22 μL ddPCR mastermix. Droplets were generated using the QX200 Droplet Generator. The generated droplet suspensions were transferred into a 96-wells plate and amplified by using a C1000 Thermal Cycler (Bio-Rad). The G+/G- PCR cycling conditions consisted of 95°C for 10 min, 40 cycles of 95°C for 30 s and 61°C for 1 min, and a final step at 98°C for 10 min. The LcS quantification conditions consisted of 95°C for 10 min, 40 cycles of 95°C for 30 s, 63°C for 1 min and 72°C for 30 s, and a final step at 98°C for 10 min. After amplification, the fluorescent signals in each single droplet were detected, measured and quantified with the QX200 Droplet Reader (Bio-Rad) and the results were processed and shown with QuantaSoft software version 1.7.4 (Bio-Rad). G+/G- -ratio and LcS copies/μL were automatically determined by the QuantaSoft software after manually setting the thresholds above the negative (signal-arm) droplets.

#### Microbiome analysis

Microbiota profiling for taxonomic classification by amplicon sequencing was carried out by Novogene Co. To this, 250 bp (paired-end) of sequence from 16S rRNA genes of distinct regions (16SV4/16SV3/16SV3-V4/16SV4-V5) were amplified using specific primers (e.g., 16S V4: 515F-806R, 18S V4: 528F-706R, 18S V9: 1380F-1510R et al.) with barcode. PCR reactions were carried out with Phusion High-Fidelity PCR Master Mix (New England Biolabs). PCR products were mixed at equal density ratios, and mixed PCR products were purified with Qiagen Gel Extraction Kit (Qiagen, Germany). Sequencing libraries were generated with NEBNext UltraTM DNA Library Prep Kit for Illumina and quantified via Qubit and Q-PCR. Sequencing was performed using the Illumina Novaseq 6000 platform.

##### Processing of sequencing data

Paired-end reads were assigned to samples based on their unique barcodes and truncated by cutting off the barcode and primer sequences. Paired-end reads were merged using FLASH (V1.2.7)^52^ (http://ccb.jhu.edu/software/FLASH/), a fast and accurate analysis tool, which was designed to merge paired-end reads when at least some of the reads overlap the read generated from the opposite end of the same DNA fragment, and the splicing sequences were called raw tags. Quality filtering on the raw tags were performed under specific filtering conditions to obtain the high-quality clean tags^53^ according to the Qiime (V1.7.0) workflow^54^ (http://qiime.org/scripts/split_libraries_fastq.html) quality controlled process.

Tags were compared to the reference database (Gold database) using the UCHIME algorithm^55^ (http://www.drive5.com/usearch/manual/uchime_algo.html) to detect chimera sequences (https://drive5.com/usearch/manual/chimeras.html) and remove chimeras^56^ to obtain the final Effective Tags. Raw sequencing yielded an average of 139,510 paired-end reads per sample. After quality filtering and chimera removal, an average of 103,565 effective tags per sample remained.

##### OTU clustering and taxonomic annotation

Sequence analysis was performed by Uparse software (Uparse v7.0.1001, http://drive5.com/uparse/)^57^ using the effective tags. Sequences with ≥97% similarity were assigned to the same OTUs. Representative sequence for each OTU was screened for further annotation. For each representative sequence, Mothur software^58^ was used to compare reads against the SSUrRNA database of SILVA Database (http://www.arb-silva.de/)for species annotation at each taxonomic rank (Threshold:0.8–1; kingdom, phylum, class, order, family, genus, species).^59^

To obtain the phylogenetic relationship of all OTUs representative sequences, the MUSCLE (Version 3.8.31, http://www.drive5.com/muscle/) aligner^60^ was used for fast comparison of sequences. OTUs abundances were normalized using a standard of sequence number corresponding to the sample with the least sequences (corresponding to 73,725 reads). Subsequent analysis of alpha diversity and beta diversity were all performed based on this output normalized data.

##### Alpha diversity

Alpha diversity was calculated to analyze complexity of biodiversity for a sample through 6 indices, including Observed-species, Chao1, Shannon, Simpson, ACE, Good-coverage. All indices in samples were calculated with QIIME (Version 1.7.0) and displayed with R software (Version 2.15.3). The following Alpha Diversity Indices were calculated:

Community richness indices: Chao - the Chao1 estimator (http://scikit-bio.org/docs/latest/generated/skbio.diversity.alpha.chao1.html#skbio.diversity.alpha.chao1); ACE - the ACE estimator (http://scikit-bio.org/docs/latest/generated/skbio.diversity.alpha.ace.html#skbio.diversity.alpha.ace); Community diversity indices:

Shannon - the Shannon index (http://scikit-bio.org/docs/latest/generated/skbio.diversity.alpha.shannon.html#skbio.diversity.alpha.shannon);

Simpson - the Simpson index (http://scikit-bio.org/docs/latest/generated/skbio.diversity.alpha.simpson.html#skbio.diversity.alpha.simpson); The index of sequencing depth:

Coverage - the Good’s coverage (https://scikit.bio/docs/dev/generated/skbio.diversity.alpha.goods_coverage.html#skbio.diversity.alpha.goods_coverage); The index of phylogenetic diversity:

PD_whole_tree - PD_whole_tree index (http://scikit-bio.org/docs/latest/generated/skbio.diversity.alpha.faith_pd.html?highlight=pd#skbio.diversity.alpha.faith_pd).

##### Beta diversity

Beta diversity analysis was used to evaluate differences of samples in species complexity. Beta diversity on both weighted and unweighted unifrac were calculated by QIIME software (Version 1.7.0). Cluster analysis was preceded by principal component analysis (PCA), which was applied to reduce the dimension of the original variables using the FactoMineR package and ggplot2 package in R software (Version 2.15.3). Principal Coordinate Analysis (PCoA) was performed to get principal coordinates and visualize from complex, multidimensional data. A distance matrix of weighted or unweighted unifrac among samples obtained before was transformed to a new set of orthogonal axes, by which the maximum variation factor is demonstrated by first principal coordinate, and the second maximum one by the second principal coordinate, and so on. PCoA analysis was displayed by WGCNA package, stat packages and ggplot2 package in R software (Version 2.15.3). Unweighted Pair-group Method with Arithmetic Means (UPGMA) Clustering was performed as a type of hierarchical clustering method to interpret the distance matrix using average linkage and was conducted by QIIME software (Version 1.7.0).

Metastat was calculated by R software. P-value was calculated by method of permutation test while q-value was calculated by method of Benjamini and Hochberg False Discovery Rate.^61^ Anosim, MRPP and Adonis were performed by R software (Vegan package: anosim function, mrpp function and adonis function). AMOVA was calculated by mothur using amova function. T_test and drawing were conducted by R software.

### Quantification and statistical analysis

Post-hoc pairwise comparisons of alpha-diversity for all group comparisons at the genus level were assessed using the Shannon index with Mann-Whitney or Kruskal-Wallis tests. The multi-testing adjustment is based on the Benjamini-Hochberg procedure (FDR). The differences in microbial composition and diversity between experimental groups were evaluated using the Bray–Curtis dissimilarity index. Permutational multivariate analysis of variance (PERMANOVA) was performed on the genera-level abundance profiles of the samples to assess the effects of location sites and interventions. The PERMANOVA and all *p* values were adjusted for false discovery rate (FDR) using the Benjamini–Hochberg procedure. Principal coordinate analyses (PCoA), Principal component analysis (PCA), partial-, and redundancy analysis (RDA) were visualized and assessed using the Canoco 5.10 software suite (Microcomputer Power, Ithaca USA) with 1000 permutations to measure significance and where applicable, corrected for covariates (histological type and patients; LcS intervention and patients). Where necessary, relative abundance values were log-transformed (Y’ = log(Y + 1000)). The RDA plot figures show the top 10 bacterial species with the highest RDA values.

Differential abundance of specific microbiota taxa in groups (tissue type, before or after LcS intervention) were also characterized using the Linear Discriminant Analysis (LDA) effect size (LEfSe) procedure (see main text), employed to classify and reduce dimensionality by identifying the optimal linear combination of taxa for the experimental variable (e.g., esophageal regions and intervention). LEfSe identifies the characteristics (organisms, clades, operational taxonomic units, genes, or functions) most likely to elucidate differences across classes by integrating traditional statistical significance tests with further assessments that account for biological consistency and effect relevance. The Kruskal-Wallis rank-sum test and effect size measurements were applied to detect differentially abundant taxa between the two groups in each comparison.

Clinical histology scoring for histologic features on formalin fixed biopsies was performed for all 23 patients pre-LcS. Histologic features including NSE and MCE presence in biopsies and inflammation scoring was performed for 19 patients pre-LcS and 17 patients post-LcS on both NSE and MCE biopsies depending on the quality of the biopsies for proper assessment. Microbiota analysis was performed on all 23 patients pre- and post-LcS. Some samples could not be included due to poor DNA quality or failed library preparation, which resulted in a total of 87 samples (NSE; 21 pre-LcS and 20 post-LcS, MCE; 23 pre-LcS and 23 post-LcS) (Table S1). ddPCR was performed for the 20 patients with good quality DNA pre- and post-LcS. A Wilcoxon signed-rank test was used to determine the significance of differences in Gram-positive/Gram-negative -ratio between baseline (day = 0) and after LcS intervention (day = 29) within each sample type group and to evaluate the difference in relative abundance of specific bacterial taxa per mm tissue between baseline and after intervention. A paired Wilcoxon signed-rank test was used to determine the difference in relative abundance of specific bacterial taxa between NSE and MCE before, and after intervention. All data were analyzed in GraphPad Prism 10. v1.2 (Dotmatics). The *p*-value <0,05 cutoff was used to determine statistical significance. In figures the following markers were used to define statistical significance: ∗*p* < 0.05; ∗∗*p* < 0,01; ∗∗∗*p* < 0.005; ∗∗∗∗*p* < 0.001.

### Additional resources

This study was registered in the Netherlands Trial Register (NL-OMON43164).

## Supplemental information

Document S1. Figures S1–S7 and Tables S2–S5

Table S1. Overview analysis performed on each case and sample

Table S6. Processed relative abundance table
