# Supplementary material for: A fermented milk drink containing Lactobacillus casei strain Shirota modulates the esophageal microbiome composition in Barrett’s esophagus
Source: iScience. 2026 Jul 22;29(8):116910. doi: 10.1016/j.isci.2026.116910 (PMC13426229; doi:10.1016/j.isci.2026.116910)
Supplement: Document S1. Figures S1–S7 and Tables S2–S5 [file mmc1.pdf]

iScience, Volume *29*

Supplemental information

A fermented milk drink containing *Lactobacillus casei* strain Shirota modulates the esophageal microbiome composition in Barrett's esophagus

Yonne Peters, Maria Laura Ferrando, Chengliang Zhou, Rene te Morsche, Britt van der Leeden, Renske Cremers, Phuc Dat Le, Leander van Dijk, Ruud W.M. Schrauwen, Adriaan C. Tan, Rachel S. van der Post, Peter van Baarlen, Peter D. Siersema, and Annemarie Boleij

Supplemental file

Content:

1. Supplemental figures
2. Supplemental tables

Supplemental Figures

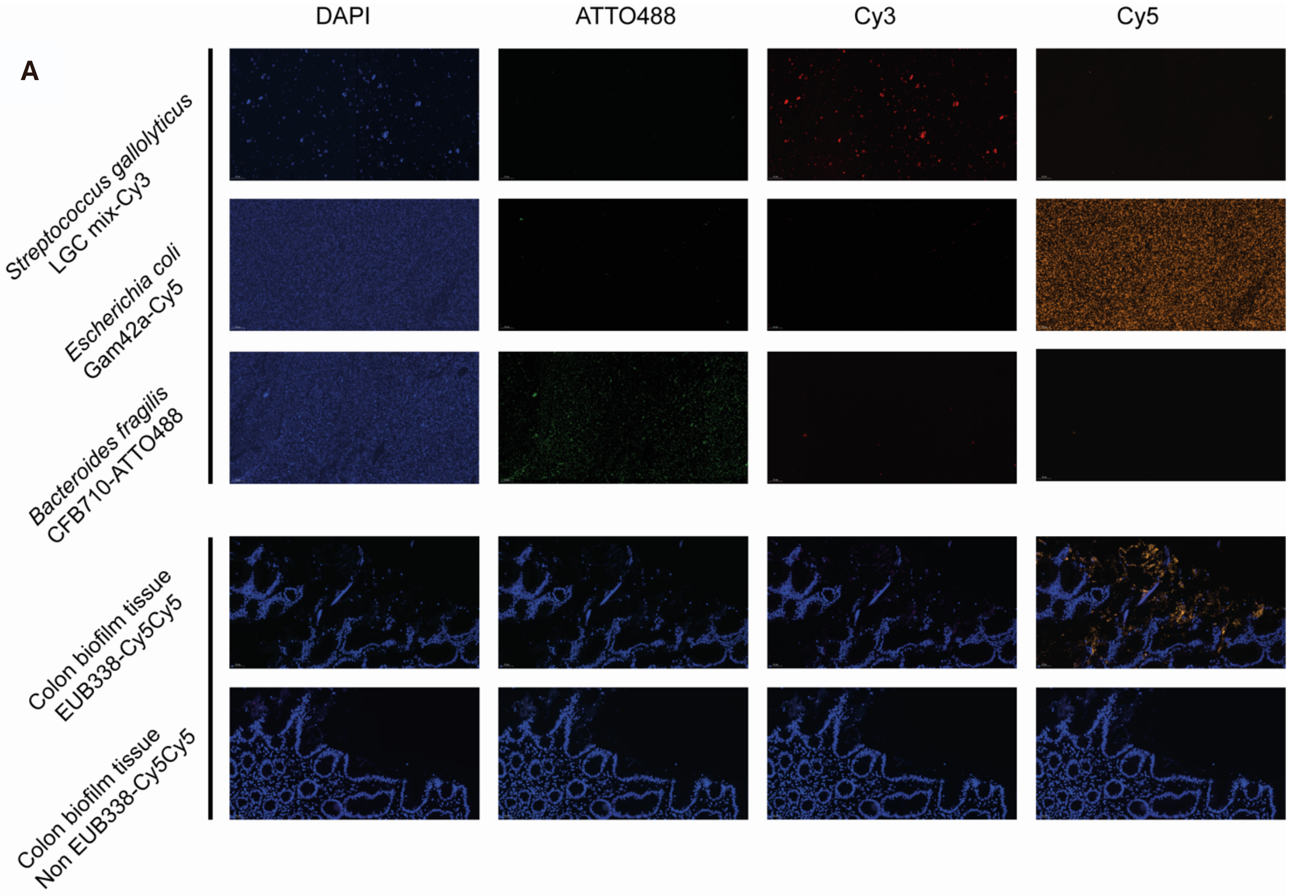

B

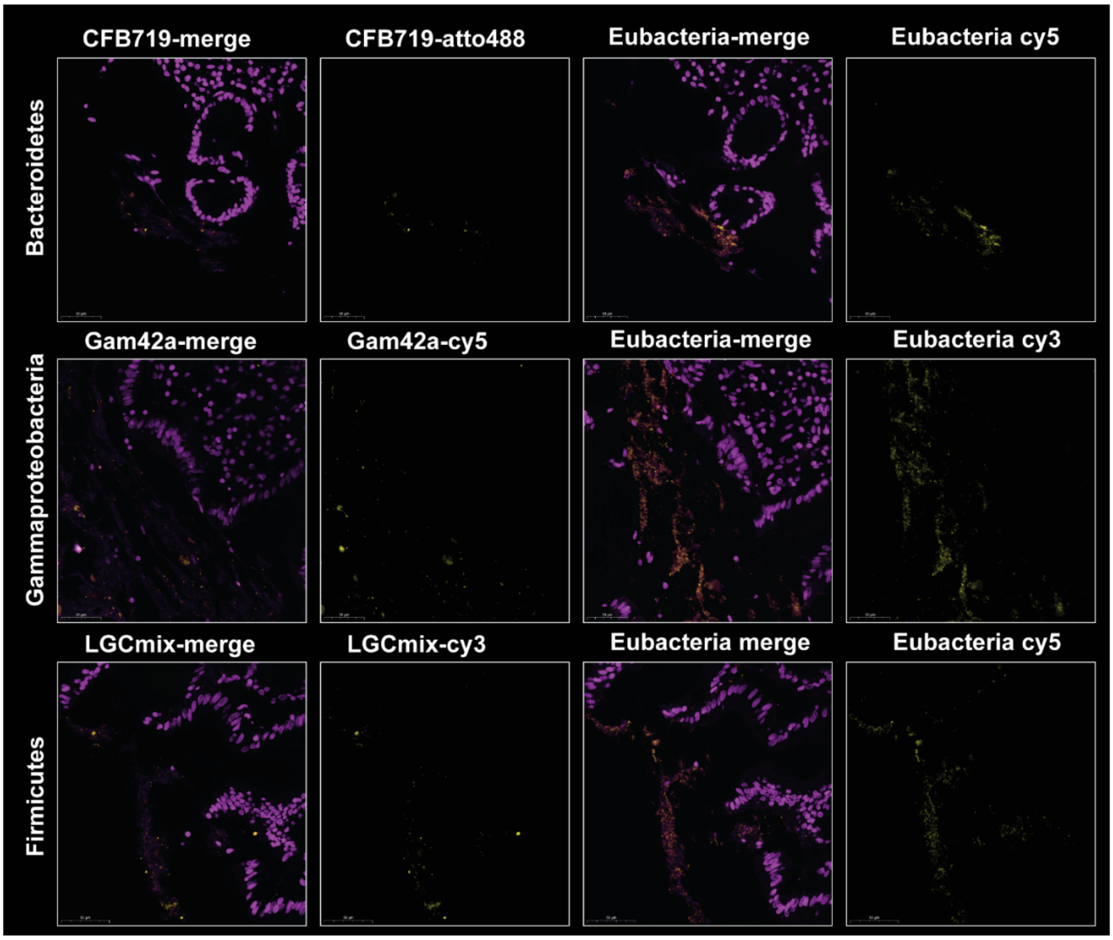

Supplemental Figure S1. Representative FISH images of control bacteria and colon tissue. [Related to Figure 2]

A) Validated FISH probes for *in situ* detection of Firmicutes (LGC-mix-cy3 (red)), Bacteroidetes (CFB719-ATTO488(green)) and Gammaproteobacteria (Gam42a (orange)) were tested on agar embedded bacteria in 4µm slices. These validated probes were selected from Probe-base [S1] and tested on *Streptococcus gallolyticus* representing Firmicutes, *Escherichia coli* representing Gammaproteobacteria and *Bacteroides fragilis* representing Bacteroidetes. All probes worked properly and for each staining a non-EUB338 control was taken along to confirm specificity of the signals. The eub338cy5cy5 probe (orange) was used to detect Eubacteria. A colon biopsy with biofilm was used as a positive control in the experiments. DAPI (blue) stains the nuclei and bacterial DNA. B) FISH images of control colon tissue. FISH probes for Firmicutes (LGC-mix-cy3), Bacteroidetes (CFB719-ATTO488) and Gammaproteobacteria (Gam42a) were tested on a control colon biopsy with a biofilm to test the feasibility of the probes on human tissue. All specific probes were simultaneously stained with EUB338 detecting Eubacteria labeled with cy5 or cy3 depending on the label of the specific probe. Bacteroidetes, Gammaproteobacteria and Firmicutes were detected in the colon biofilm. The DNA is stained with DAPI and visible in the merged image in purple. The bacterial signal in each image is depicted in yellow. Scalebars in the left corner are 100 µm for all images in A and 50µm in length for all images in B.

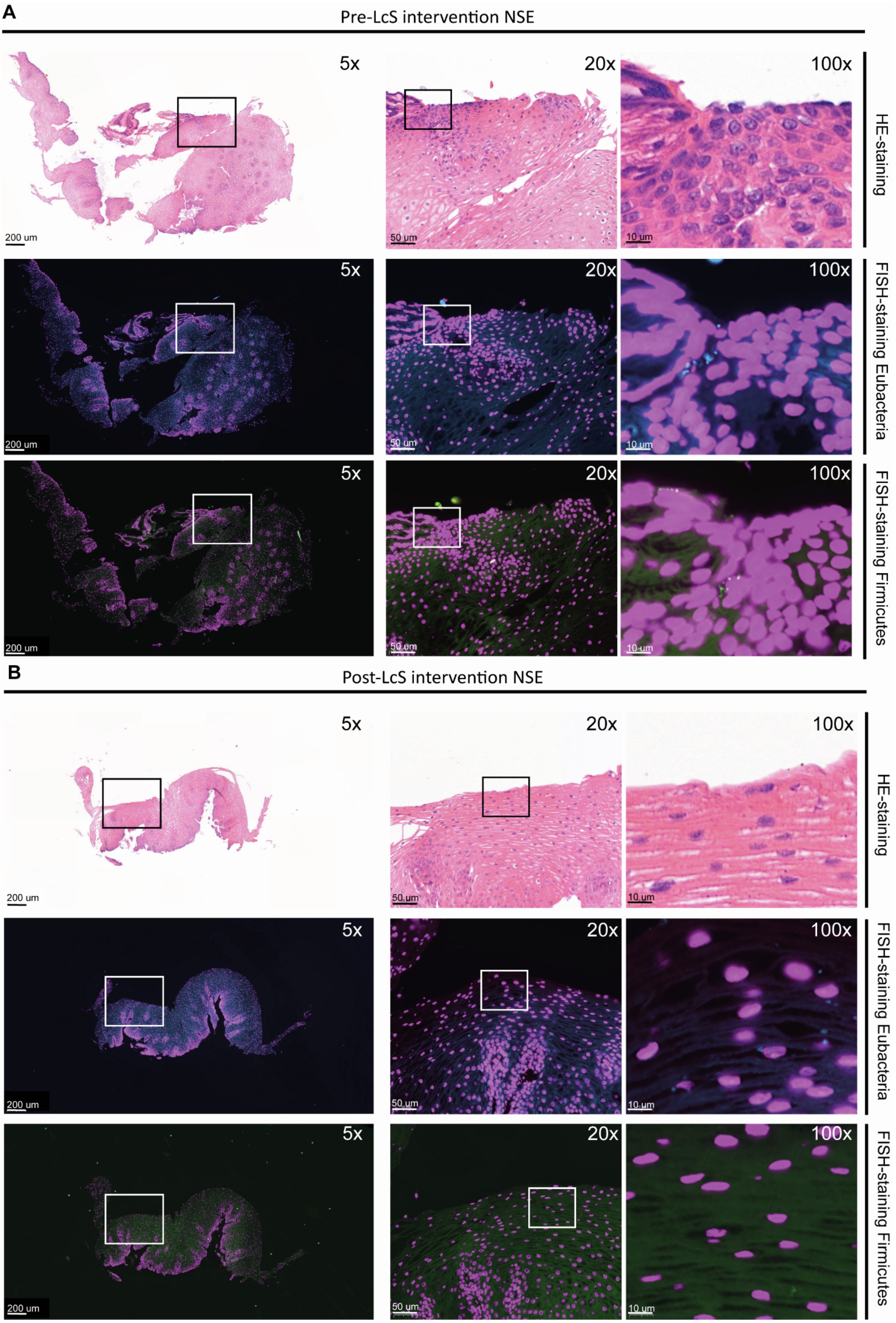
A
Pre-LcS intervention NSE
5x
20x
100x
200 um
50 um
10 um
HE-staining
FISH-staining Eubacteria
FISH-staining Firmicutes
B
Post-LcS intervention NSE

Supplemental figure S2. *In situ* bacterial detection of Eubacteria and Firmicutes. [Related to Figure 2]

A) NSE biopsies pre-LcS and B) post-LcS intervention. Eubacteria are depicted in cyan, Firmicutes are depicted in green and DAPI is depicted in Magenta. The length of the scale bars is indicated at 5x 200µm, at 20x 50µm and at 100x 10µm.

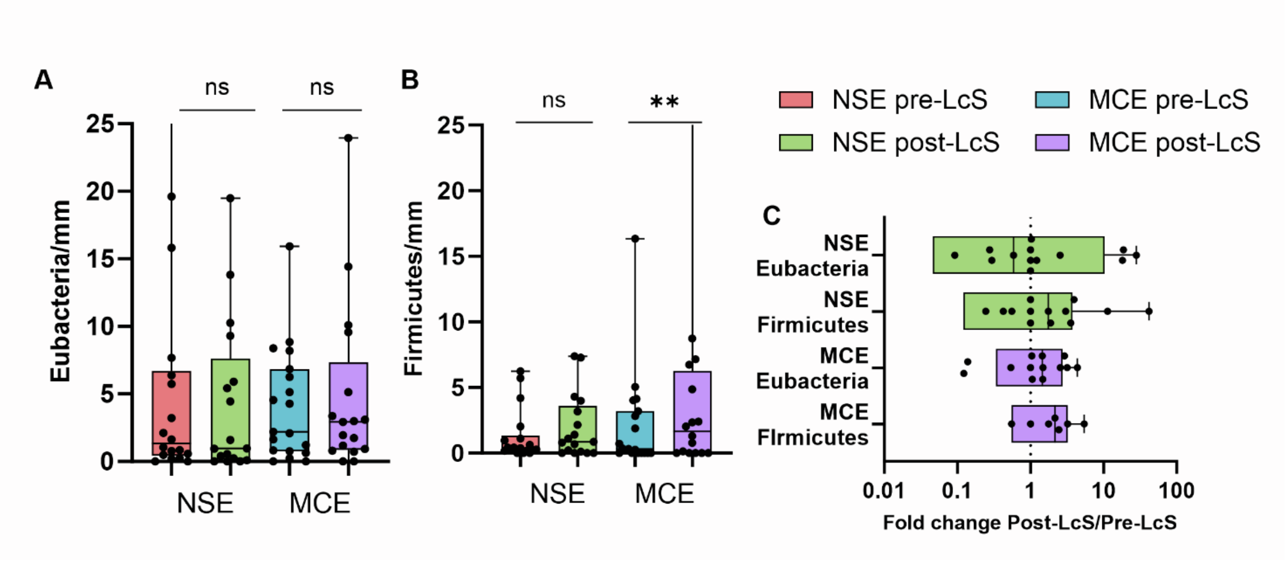

Supplemental figure S3. Eubacteria, Firmicutes signals detected per mm apical region in NSE and MCE tissue biopsies [Related to Figure 2]

A) No statistically significant difference was observed in the number of Eubacteria signals per mm in both MCE (median 2.19, and 2.93), and NSE biopsies (median 1.35 and 0.96) pre- and post-LcS, respectively. B) A significant increase in Firmicutes signals post-LcS was observed per mm apical region for MCE biopsies (median 0.32 vs 1.67; ** $p < 0{,}01$ Wilcoxon Signed Rank test), but no significant increase in Firmicutes post-LCS was observed in NSE biopsies (median 0.4 vs 0.86). C) the ratio of bacteria per mm tissue post- versus pre-LcS is depicted on the x-axis, a ratio of 1 (dotted line) means no difference post- versus pre-LcS, a positive ratio means an increase post-LcS, a fold change of >2 was observed for Firmicutes in MCE biopsies in 5 out of 7 paired biopsies. All images display median, with IQR and min-max.

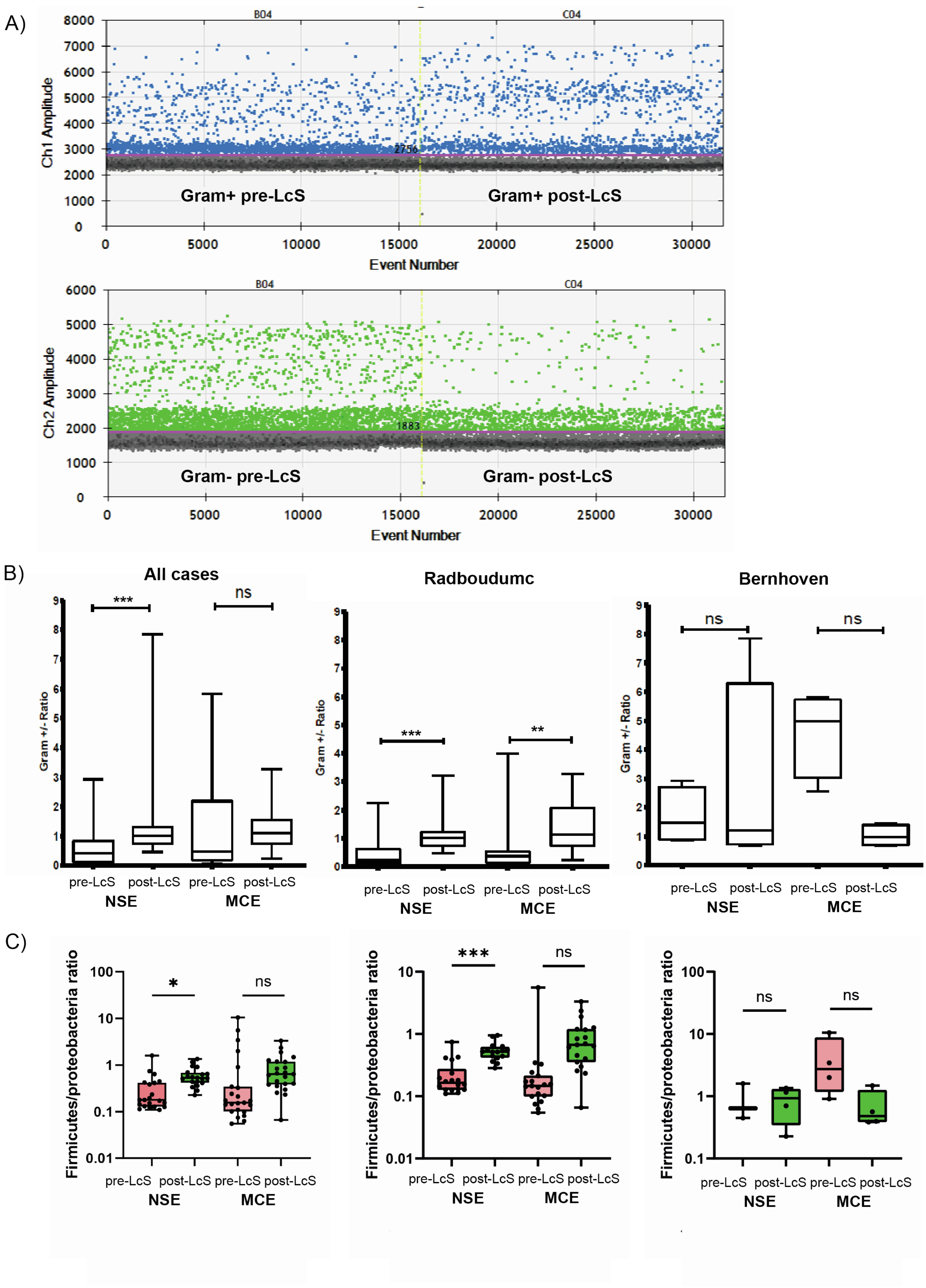
A)
B04
C04
Ch1 Amplitude
8000
7000
6000
5000
4000
3000
2000
1000
0
2756
Gram+ pre-LcS
Gram+ post-LcS
0
5000
10000
15000
20000
25000
30000
Event Number
Ch2 Amplitude
6000
5000
4000
3000
2000
1000
0
1883
Gram- pre-LcS
Gram- post-LcS
Event Number
B)
All cases
Radboudumc
Bernhoven
Gram +/- Ratio
***
ns
**
pre-LcS
post-LcS
NSE
MCE
C)
Firmicutes/proteobacteria ratio
100
10
1
0.1
0.01
*
***
ns

Supplemental Figure S4. Gram-positive and Gram-negative ratios based on ddPCR and relative abundance. [Related to section Gram-positive to Gram-negative ratio in results]

A) Example of Gram+ and Gram- bacteria by ddPCR detection in MCE biopsies pre- and post-LcS intervention. A notable increase in Gram-positive bacteria and decrease in Gram-negative bacteria is observed. Blue dots on channel 1 (y-axis) represent Gram+ bacteria; Green dots on channel 2 (y-axis) represents Gram- bacteria (Gram-); Event number on the x axis showcase the total droplet count analyzed. Amplitude represented by the y-axis accounts for total signal per droplet. Droplets above the threshold line (purple line) were determined as positive result. B) Changes in Gram+ /Gram- ratio (y axis) of all patients (n=20), only Radboudumc patients (n=16) and only Bernhoven patients (n=4) pre- and post-LcS intervention according to 16S rRNA ddPCR analyses. A significant increase of 0.42 to 1.02 in Gram+ /Gram- ratio for NSE was observed (*** p=0.003, Wilcoxon signed-rank test), and a non-significant increase of 0.48 to 1.11 for MCE biopsies. Separate analysis of the Radboudumc patients (n=16) showed a significant increase from 0.21 to 1.02 (*** p=0.004) for NSE and 0.36 to1.12 (** p=0.008) for MCE post-LcS intervention. The Bernhoven patients (n=4) subgroup was too small to test for statistical significance in separate analysis. C) Gram+ to Gram- ratio calculated based on the relative abundance of the phyla Firmicutes and Proteobacteria. Similar to the results of the ddPCR a significant increase in Firmicutes to Proteobacteria ratio was seen post-LcS intervention in NSE biopsies (* p=0.010, Wilcoxon signed-rank test), and non-significant increase in MCE biopsies. Separate analysis of the Radboudumc patients (n=16) showed a significant increase in both NSE (*** p=0.0009) and MCE biopsies (** p=0.0012) of Firmicutes to proteobacteria ratio post-LcS. All box-plots show median with IQR and min-max values.

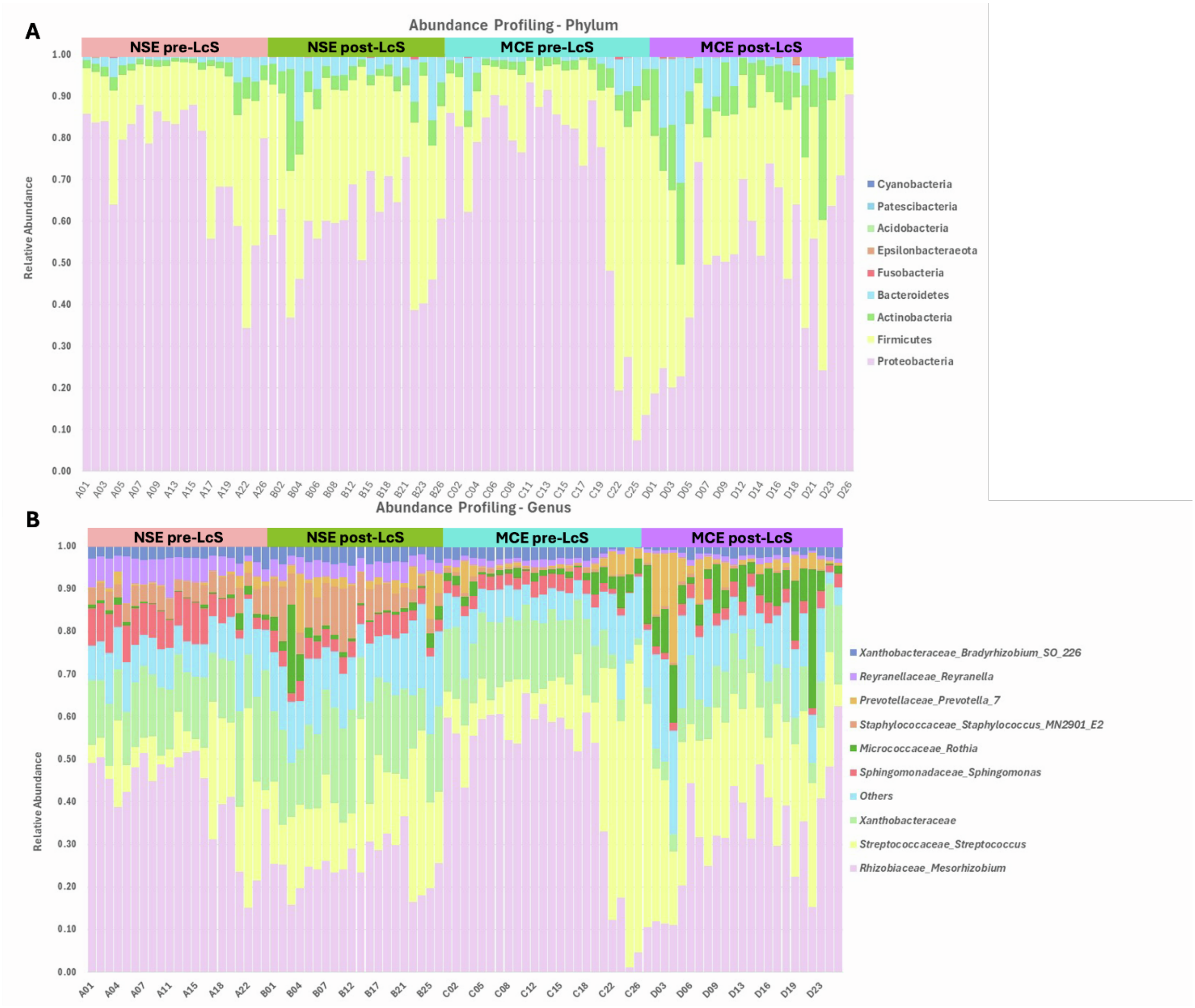

Supplemental Figure S5. Relative abundance of bacterial taxa at Phylum (A) and Genus (B) level in NSE and MCE samples pre- and post-LcS [Related to Figure 3]

Phylogenetic profiles were more similar within individuals than between the two sampled anatomical locations with either normal healthy (squamous (NSE)) or BE (columnar) morphology (MCE). Microbial profile of top ten taxa at phylum and genus level in patients affected by BE in two different esophageal locations (NSE (orange) and MCE (green)) before (pre-LcS) and after intervention (post-LcS) with LcS is shown at phylum (A) and genus (B) level. Individuals are grouped for the four experimental categories.

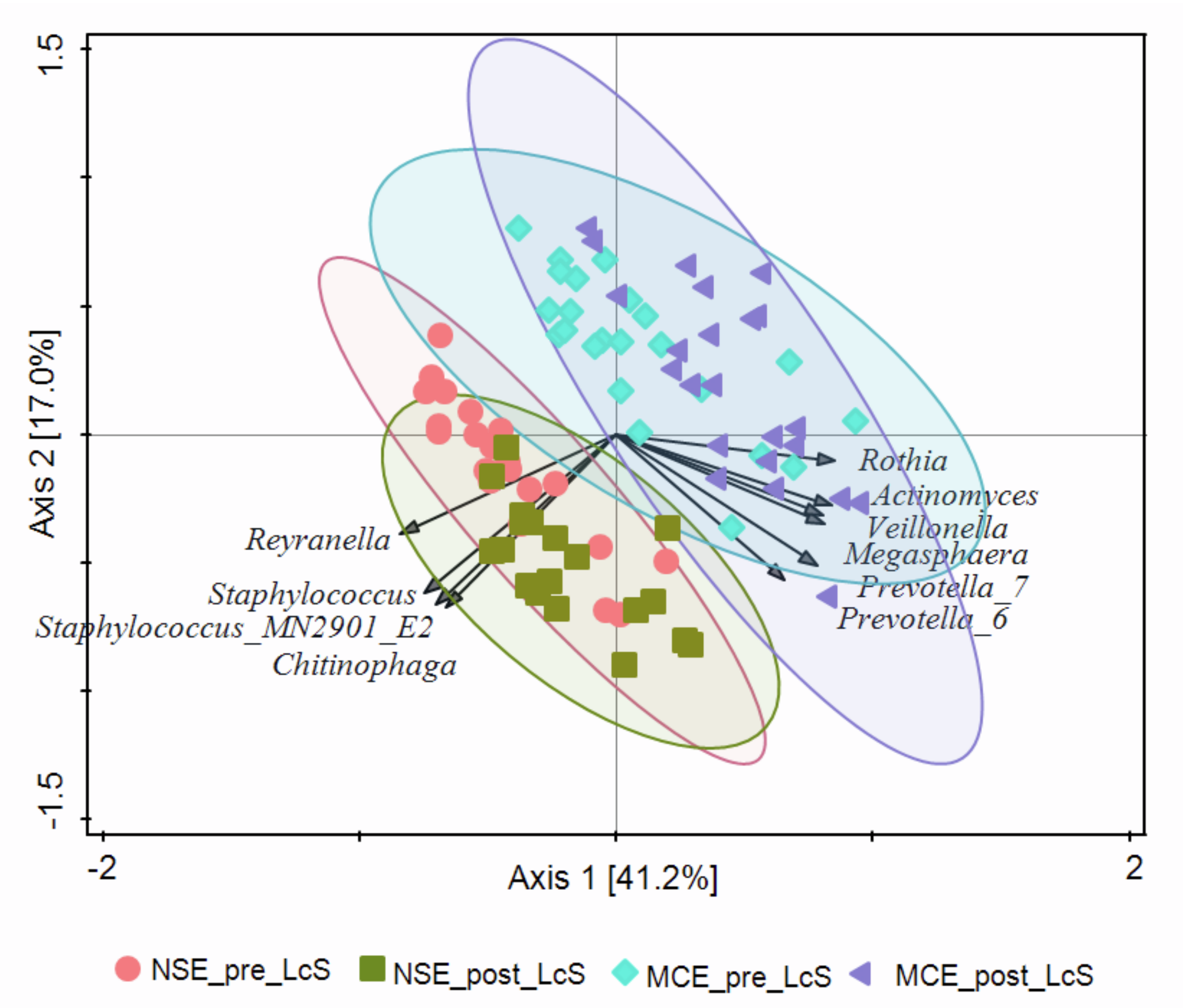

Supplemental Figure S6. Principal component analysis of microbiota composition.[Related to Figure 4]

Principal component analysis (PCA) visualizes differences in microbiota composition at genus level associated with sampled esophageal regions and pre- or post-LcS intervention sampling time points. PCA was performed at the genus level using individual samples, which are represented by symbols for each experimental group. The top 10 genera with the highest contributions to the principal components (PC) are plotted as arrows. Ellipses represent 95% confidence intervals for the groupings. The percentages along the axes indicate the proportion of variation explained by the PC; both axes explain nearly 60% of the total variation in the data.

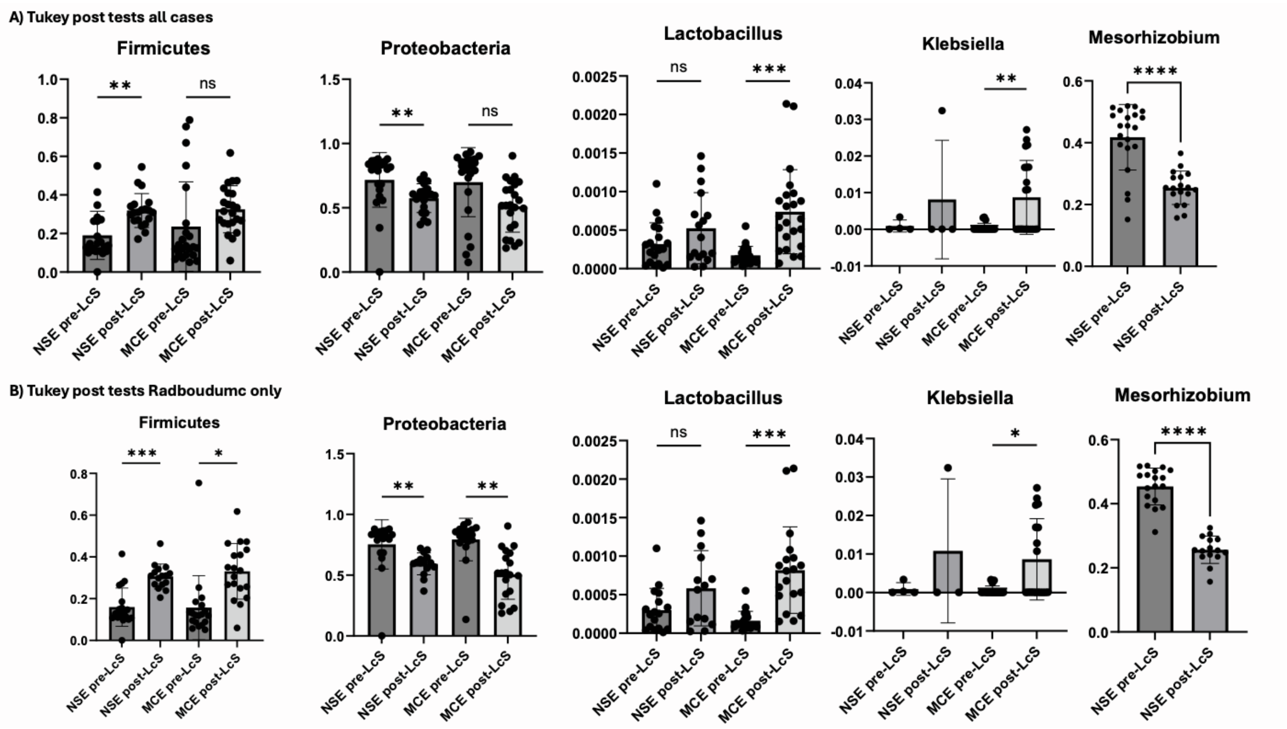

Supplemental Figure S7. Tukey's post hoc test of relative abundance of taxa pre- and post LcS in NSE and MCE biopsies. [Related to Figure 5]

A) Relative abundance plots of all cases (including Bernhoven hospital; n=23), and B) of patients from the Radboudumc hospital only (n=19). Relative abundance plots are plotted foreach group separately (NSE pre-LcS, NSE post-LcS, MCE pre-LcS and MCE post-LcS). *$p<0.05$, **$p<0.01$, ***$p<0.005$, **** $p<0.001$. Bar plots show the average RA with SD.

Supplemental Tables

Supplemental Table S2. Fluorescence *in situ* hybridization probes [Related to STAR Methods]

	Target	Oligo Name-Label	Target sequence (5'-3')
Panel mixture	*Bacteroidetes*	CFB719-Atto488	AGCTGCCTTCGCAATCGG
	Firmicutes	LGC354A-Cy3<br>LGC354B-Cy3<br>LGC354C-Cy3	TGGAAGATTCCCTACTGC<br>CGGAAGATTCCCTACTGC<br>CCGAAGATTCCCTACTGC
	Gammaproteobacteria	Gam42a-Cy5	GCCTTCCCACATCGTTT
All bacteria	Universal Eubacteria	EUB338-Cy5Cy5	GCTGCCTCCCGTAGGAGT
Negative control	complimentary to universal Eubacteria	Non-EUB338-Cy5Cy5	ACTCCTACGGGAGGCAGC

Supplemental Table S3. Post-hoc pairwise comparisons of alpha diversity [Related to Figure 4]

**Experimental groups**	**Statistics**	**p-values**	**FDR**
NSE vs MCE	1468.0	4.00E-06	**4.00E-06**
pre_LcS vs post_LcS	297.0	5.32E-09	**5.32E-09**
NSE_pre_LcS vs NSE_post_LcS	40	1.49E-06	**2.99E-06**
NSE_pre_LcS vs MCE_pre_LcS	439	4.19E-07	**1.26E-06**
NSE_pre_LcS vs MCE_post_LcS	185	1.90E-01	1.90E-01
NSE_post_LcS vs MCE_pre_LcS	457	1.46E-11	**8.74E-11**
NSE_post_LcS vs MCE_post_LcS	387	6.14E-05	**7.37E-05**
MCE_pre_LcS vs MCE_post_LcS	69	4.82E-06	**7.23E-06**

Post-hoc pairwise comparisons of alpha-diversity for all group comparisons at the genus level. Significant ($p < 0.05$) results are in bold.

Supplemental Table S4: Pairwise PERMANOVA [related to Figure 4]

Experimental groups	F-value	R-square	p-values	FDR
NSE vs MCE	17.351	0.16953	0.001	**0.001**
pre_LcS vs post_LcS	13.133	0.13383	0.001	**0.001**
MCE_pre_LcS vs MCE_post_LcS	7.2402	0.1413	0.008	**0.008**
NSE_pre_LcS vs MCE_pre_LcS	7.1909	0.14618	0.002	**0.0024**
NSE_pre_LcS vs NSE_post_LcS	14.429	0.27007	0.001	**0.0015**
NSE_pre_LcS vs MCE_post_LcS	25.568	0.3784	0.001	**0.0015**
NSE_post_LcS vs MCE_pre_LcS	13.309	0.24506	0.001	**0.0015**
NSE_post_LcS vs MCE_post_LcS	17.454	0.2986	0.001	**0.0015**

Summary of multivariate pairwise PERMANOVA testing for statistical differences between esophageal regions and interventions. Significant ($p < 0.05$) results are in bold.

Supplemental table S5: Rank correlation analysis using Linear discriminant analysis effect size (LEfSe) [Related to Figure 5]

A) at the genus level between the esophageal regions normal squamous (NSE) and metaplastic columnar epithelium (MCE) B) at the genus level between Pre and Post-LcS intervention.

Table S5A

**Taxa (Phylum)**	**P-values[#]**	**FDR[§]**	**NSE***	**MCE***	**LDA score^**
Actinobacteria	9.77E-03	2.20E-02	38135	62894	4.09
Fusobacteria	2.54E-02	3.81E-02	1333.7	6815.3	3.44
Patescibacteria	2.05E-04	6.15E-04	253.38	663.69	2.31
Epsilonbacteraeota	2.21E-02	3.81E-02	765.91	974.88	2.02
Cyanobacteria	1.49E-15	6.71E-15	366.38	54.789	-2.2
Acidobacteria	3.53E-16	3.18E-15	1249.6	224.41	-2.71
**Taxa (Genus)**	**P-values**	**FDR**	**NSE**	**MCE**	**LDA score**
Staphylococcus_sp__clone_MN2901_E2	1.07E-15	2.30E-14	5448.6	59874	-4.43
Reyranella	3.00E-15	4.30E-14	11567	44842	-4.22
Sphingomonas	1.86E-07	6.41E-07	27989	53864	-4.11
Staphylococcus	1.07E-15	2.30E-14	695.67	14118	-3.83
Bradyrhizobium_sp__SO_226	2.47E-05	6.85E-05	21942	31528	-3.68
Chitinophaga	1.07E-15	2.30E-14	1689.8	11054	-3.67
Pseudolabrys	2.13E-15	3.67E-14	2900.2	8318.9	-3.43
Hyphomicrobium	9.84E-05	2.49E-04	4519.1	6573	-3.01
Bradyrhizobium	7.14E-13	4.73E-12	873.85	1556.2	-2.53
Cutibacterium	1.91E-12	1.18E-11	389.01	1036.2	-2.51
Arthrobacter	1.75E-08	6.84E-08	0	465.65	-2.37
Hydrogenophilus	2.59E-07	8.57E-07	44.427	354.66	-2.19
Anoxybacillus	1.14E-13	9.78E-13	19.773	245.84	-2.06
Comamonas	3.17E-09	1.36E-08	3.9851	217.15	-2.03
Rhodoplanes	3.61E-08	1.35E-07	62.544	265.24	-2.01
Caulobacter	5.53E-11	2.97E-10	97.773	273.7	-1.95
Hephaestia	4.33E-14	4.13E-13	87.631	251.63	-1.92
Corynebacterium_1	1.07E-03	2.36E-03	55.872	175.53	-1.78
Faecalibacterium	5.97E-08	2.14E-07	0.28439	97.674	-1.7
Bryobacter	1.24E-09	5.59E-09	15.144	107.91	-1.68
Streptomyces	2.68E-06	8.24E-06	203.1	297.19	-1.68
Rudaea	3.91E-04	9.10E-04	170.38	259.65	-1.66
Edaphobacter	7.39E-16	2.3E-14	4.3469	87.947	-1.63
uncultured_cyanobacterium	6.10E-10	2.91E-09	28.751	107.68	-1.61
Pseudomonas	1.83E-04	4.50E-04	200.55	275.29	-1.58
Legionella	1.92E-10	9.73E-10	36.03	103.2	-1.54
Roseomonas	3.66E-05	9.54E-05	48.846	0	1.41
TM7_phylum_sp__oral_clone_DR034	6.50E-04	1.47E-03	578.74	252.82	2.21
Ralstonia	1.21E-08	4.96E-08	678.28	313.35	2.26
Pseudonocardia	1.35E-03	2.83E-03	1265.6	889.81	2.28
Pseudaminobacter	1.20E-03	2.57E-03	1551.3	1031.6	2.42
Acinetobacter	3.39E-13	2.65E-12	1149.2	29.247	2.75
Sphingobacterium	3.37E-06	9.99E-06	1809.2	1.0876	2.96
Granulicatella	3.46E-05	9.29E-05	5226.1	2343.4	3.16

Actinomyces	2.56E-04	6.11E-04	6062	2724.3	3.22
Klebsiella	2.56E-11	1.47E-10	4264.2	793.29	3.24
Rothia	6.27E-07	2.00E-06	49322	16689	4.21

Table S5B

Taxa (Phylum)	P-values	FDR	pre_LcS	post_LcS	LDA score
Firmicutes	2.49E-05	7.47E-05	220070	311060	4.66
Actinobacteria	8.37E-07	7.53E-06	30354	72255	4.32
Bacteroidetes	8.03E-05	1.81E-04	31658	56680	4.1
Epsilonbacteraeota	1.27E-02	1.91E-02	464.76	1289.4	2.62
Patescibacteria	4.70E-04	8.46E-04	288.38	654.89	2.27
Fusobacteria	3.72E-02	4.79E-02	6709.9	1789.1	-3.39
Proteobacteria	5.60E-06	2.52E-05	709610	555320	-4.89
**Taxa (Genus)**	**P-values**	**FDR**	**pre_LcS**	**post_LcS**	**LDA score**
Mesorhizobium	8.48E-07	4.34E-05	285530	449910	-4.91
Afipia	1.01E-06	4.34E-05	2247.7	3680.2	-2.86
Pseudaminobacter	1.74E-05	3.74E-04	976.08	1629.1	-2.52
Abiotrophia	5.61E-04	2.84E-03	200.62	107.59	1.68
Stomatobaculum	2.88E-03	1.12E-02	567.6	376.48	1.98
TM7_phylum_sp__oral_clone_DR034	3.87E-04	2.08E-03	570.48	283.12	2.16
Candidatus_Ancillula	7.51E-04	3.40E-03	614.11	261.68	2.25
Lachnoanaerobaculum	4.44E-05	5.32E-04	724.76	347.59	2.28
Lactobacillus	7.60E-05	6.42E-04	614.49	224.54	2.29
Leptotrichia	9.02E-04	3.88E-03	1300	813.08	2.39
Bifidobacterium	9.74E-05	6.42E-04	1282.7	346.24	2.67
Megasphaera	6.04E-04	2.89E-03	2252.5	1092.2	2.76
Haemophilus	2.19E-05	3.76E-04	2313.8	1132.5	2.77
Atopobium	6.81E-06	1.95E-04	3728.9	1469.8	3.05
Prevotella_6	9.41E-05	6.42E-04	4026.7	1361.5	3.13
Granulicatella	3.18E-05	4.56E-04	5445.6	2325.5	3.19
Actinomyces	5.72E-05	5.47E-04	6254.7	2763.6	3.24
Gemella	1.68E-03	6.89E-03	17223	12315	3.39
Veillonella	1.05E-04	6.42E-04	23825	9912.1	3.84
Prevotella_7	4.95E-05	5.32E-04	38655	12967	4.11
Rothia	2.37E-04	1.36E-03	52546	15763	4.26
Streptococcus	9.41E-05	6.42E-04	224240	157580	4.52

Kruskal-Wallis rank-sum test

§ Adjusted for false discovery rate (FDR) using the Benjamini–Hochberg procedure

* Read counts

^ Linear Discriminant Analysis (LDA) scores >2.5 or <−2.5 and an FDR-corrected $p < 0.05$ were considered significant.

Supplemental references

S1. Greuter, D., A. Loy, M. Horn, and T. Rattei, *probeBase--an online resource for rRNA-targeted oligonucleotide probes and primers: new features 2016*. Nucleic Acids Res, 2016. **44**(D1): p. D586-9.
